# Association of folate and vitamin B12 imbalance with adverse pregnancy outcomes among 11,549 pregnant women: An observational cohort study

**DOI:** 10.3389/fnut.2022.947118

**Published:** 2022-07-25

**Authors:** Xiaosong Yuan, Xiaoya Han, Wenbo Zhou, Wei Long, Huiyan Wang, Bin Yu, Bin Zhang

**Affiliations:** ^1^Department of Medical Genetics, Changzhou Maternal and Child Health Care Hospital, Changzhou Medical Center, Nanjing Medical University, Changzhou, China; ^2^Department of Obstetrics and Gynecology, Changzhou Medical Center, Changzhou Maternal and Child Health Care Hospital, Nanjing Medical University, Changzhou, China

**Keywords:** folate, vitamin B12, birth weight, pregnancy complications, birth outcome, large-for-gestational age

## Abstract

**Objective:**

This study aimed to evaluate maternal serum levels of folate, vitamin B12, and their ratio on admission for labor and determine whether an imbalance between folate and vitamin B12, represented by a higher or lower serum folate to vitamin B12 ratio (SFVB12R), was associated with adverse pregnancy outcomes.

**Methods:**

A retrospective cohort study of 11,549 pregnant women attending a district specialized hospital and who had serum folate (SF) and serum vitamin B12 (SVB12) levels measured at delivery was performed. The levels of SF, SVB12, and SFVB12R were defined as high (>95th percentile), normal (5–95th percentile), and low (<5th percentile). Information on pregnancy outcomes was retrieved from medical records. Linear regression was performed to examine the association of abnormal SF, SVB12, and SFVB12R levels with fetal growth indicators. Logistic regression was applied to estimate the association between abnormal SF, SVB12, and SFVB12R levels and pregnancy outcomes.

**Results:**

Lower SF levels were associated with higher risks of intrahepatic cholestasis of pregnancy (ICP, OR 1.58; 95% CI 1.15–2.17), pre-eclampsia (PE, OR 1.89; 95% CI 1.28–2.81), and a lower risk of gestational diabetes mellitus (GDM, OR 0.40; 95% CI 0.23–0.70), whereas higher SVB12 levels were associated with a higher risk of ICP (OR 2.22; 95% CI 1.67–2.96), PE (OR 1.69; 95% CI 1.04-2.74), and GDM (OR 1.62; 95% CI 1.24–2.11). A higher SFVB12R increased birthweight (β 60.99; 95% CI 29.52–92.45) and was associated with a higher risk of large-for-gestational-age (LGA) newborns (OR 3.08; 95% CI 1.63–5.83); a lower SFVB12R decreased birthweight (β −43.81; 95% CI −75.62, −12.00) and was associated with a lower risk of LGA newborns (OR 0.75; 95% CI 0.56–1.00), and with higher risks of ICP (OR 2.03; 95% CI 1.54–2.67) and pregnancy-induced hypertension (PIH, OR 1.81; 95% CI 1.09–3.00).

**Conclusion:**

An imbalance between folate and vitamin B12, represented by a higher or lower SFVB12R before delivery, was significantly associated with adverse pregnancy outcomes (ICP/PIH/LGA).

## Introduction

Adverse pregnancy outcomes, such as gestational diabetes mellitus (GDM), pre-eclampsia (PE), and infants born small for gestational age (SGA) and preterm birth (PTB) may complicate more than 50% of all pregnancies and seriously impair maternal and fetal health in the short- and long-term (1). GDM affects more than 10% of pregnancies and a history of GDM is associated with a 40% increased risk of subsequent type 2 diabetes and cardiovascular disease (2). PE occurs in ~3–8% of all pregnancies and women with a history of PE have increased risks of metabolic syndrome, central obesity, and high blood pressure by 23, 36, and 53% later in life, respectively (3). In addition, accumulating evidence supports an increased metabolic risk among children born with PTB or SGA (4). Effective prediction and intervention of adverse pregnancy outcomes can not only improve pregnancy health but also improve child health.

Folate (vitamin B9) and vitamin B12 are important micronutrients required for DNA synthesis and epigenetic regulation (5). Fetal growth and development require adequate folate and vitamin B12, especially during mid and late pregnancy (6). Inadequate status of maternal folate and vitamin B12 during pregnancy are associated with poor perinatal outcomes (7). Folate supplementation is routinely recommended during early pregnancy to prevent neural tube defects. However, the importance of vitamin B12 supplementation during pregnancy is usually unrecognized although the insufficient status of vitamin B12 in pregnant women is widely prevalent, especially in developing countries. There is an increasing attention on the relationship between maternal B vitamin imbalance and pregnancy complications in the past decade. Several studies to date have reported inconsistent associations between maternal B vitamin imbalance, represented by high folate, low vitamin B12 status, and GDM risk (8–12). In addition, only a few reports have been published on the associations between B vitamin imbalance during pregnancy and adverse birth outcomes (13). In addition, to our knowledge, no studies to date have examined the relationship between another maternal B vitamin imbalance, defined as low folate and high vitamin B12 status, and adverse pregnancy outcomes. Therefore, in this retrospectively observational study, we aimed to investigate the association among maternal abnormal status of folate, vitamin B12, and their ratio on admission for labor with adverse pregnancy outcomes in the Chinese population.

## Materials and methods

### Study population and data collection

**Figure 1:**
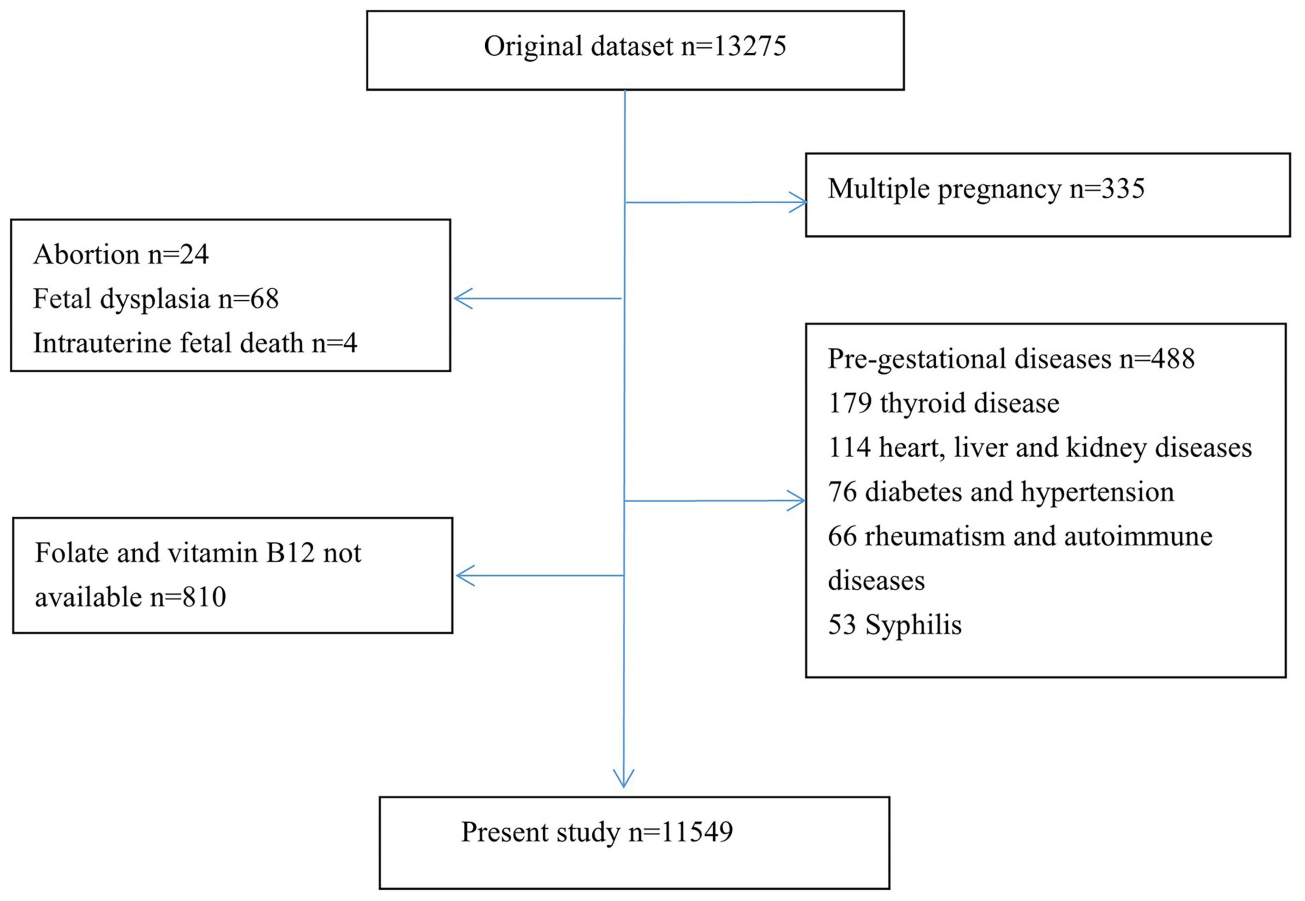
Study flow diagram.

A retrospective cohort study was performed at Changzhou Maternal and Child Health Care Hospital Affiliated with Nanjing Medical University from April 2016 to March 2017 in Jiangsu, eastern China. The study was approved by the ethics committee of the hospital (no. ZD201803), and informed consent was provided for eligible participants. Participants with a singleton pregnancy were included if they had available medical records and complete laboratory tests on admission for labor, live birth, and no fetal anomalies, and participants were excluded from the analytic data set if they used illicit drugs or alcohol or smoked during pregnancy; or had major pre-gestational diseases, including diabetes mellitus type 1 or 2, chronic hypertension, heart, liver, or kidney disease, thyroid disease, immune rheumatic disease, or syphilis. All pregnant women with adverse pre-gestational diseases, multiple pregnancies, birth of congenital malformation, and no serum folate (SF) levels or serum vitamin B12 (SVB12) levels were excluded from this cohort (*n* = 1,726). A total of 11,549 mother-and-singleton-newborn pairs were eligible for the analytic data set (Figure 1). Baseline characteristics regarding maternal age, gravidity, parity, body mass index (BMI), and blood pressure (BP) on hospital admission, gestational age before delivery, and pregnancy complications and their newborns (sex, birth length, and birthweight) were collected from the hospital records. Maternal blood samples were collected on the admission day for delivery. SF and SVB12 levels were investigated routinely and obtained from the laboratory information system of the hospital. SF and SVB12 levels were examined using chemiluminescent immunoassay using an automated analyzer (UniCel DxI 800 Access, Beckman Coulter Inc., USA). According to the manufacturer's instructions, the detection limits were <2 μg/L for SF and <50 ng/L for SVB12, and the normal references of SF and SVB12 for the healthy population were 5.9–24.8 μg/L and 180–914 ng/L. However, there is no specific reference intervals for pregnant women on admission for labor. We calculated the reference intervals of SF, SVB12 levels, and their ratios by using non-parametric methods (bilateral non-parametric 90% reference range) and defined the 5th and 95th percentiles as the lower and upper reference limits recommended by the manufacturer's instructions.

### Definition of adverse pregnancy outcomes

Adverse pregnancy outcomes included pregnancy complications [GDM, intrahepatic cholestasis of pregnancy (ICP), PE, and pregnancy-induced hypertension (PIH)] and unfavorable perinatal outcomes [(PTB, SGA, and large-for-gestational-age (LGA)]. The complications were defined according to a previous report (14). Delivery before 37 weeks of gestation was diagnosed as PTB (15). Based on a Chinese reference curve of birthweight reported in the previous report, SGA or LGA was defined as a newborn below the 10th percentile or above the 90th of birthweight for specific gestational age (16).

### Statistical analysis

Empower Stats software version 2.0 (X&Y solutions inc., USA) was used for the data analysis. In this study, categorical variables were expressed as *N* (%), and non-normally and normally distributed continuous variables were expressed as median (interquartile range, IQR) and mean ± standard deviation (SD), respectively. To compare differences in the characteristics of mother-and-singleton-newborn pairs between different categories of maternal SF, SVB12 levels, and their ratio, parametric and non-parametric tests (ANOVA and Kruskal–Wallis test) for normally and non-normally distributed continuous variables and chi-square tests for categorical variables were used.

SF, SVB12 levels, and their ratio according to different perinatal outcomes were compared by non-parametric tests. In addition, general linear analyses were applied to explore the association between fetal growth indices (birthweight, birth length, and gestational age) and maternal SF, SVB12 levels, and their ratios; logistic regression models were applied to analyze the associations of maternal SF, SVB12 levels, and their ratios with pregnancy complications and perinatal outcomes. In the adjusted models, maternal age, BMI, gravidity, parity, blood pressure, GDM, ICP, PE, PIH, fetal sex, and gestational age were regarded as confounding factors. Moreover, smooth curve fitting analysis was used to describe the potential nonlinear relations between continuous SF, SVB12, SFVB12R, and the risk of pregnancy outcomes.

## Results

### Demographic characteristics of participant

The baseline demographic characteristics of the study population according to different categories of maternal SF, SVB12, and serum folate to vitamin B12 ratio (SFVB12R) were shown in Tables 1–3. The 5th and 95th percentiles for SF, SVB12, and SFVB12R were 4.25 and 24.20 μg/L, 106 and 390 ng/L, and 20.47 and 124.74, respectively. Overall, the mean maternal age at the time of delivery was 28.6 years (range 18–47), and the mean prenatal BMI was 27.3 ± 3.4 kg/m^2^. Of the 11,549 women, 60.0% (6,933) were nulliparous, 8.4% (965) were complicated with GDM, 6.2% (711) with ICP, 3.4% (394) with PE, and 2.1% (245) with ICP. Among these single gestation live births, 6.8% (790) were classified as PTB, 8.8% (1,022) as SGA, and 15.5% (1,791) as LGA. With the elevation of SFVB12R categories (<5th percentile, 5–95th percentile, and >95th percentile), significant increasing trends were found for maternal age (28.0 vs. 28.6 vs. 29.3 years, *P* < 0.01), BMI (26.6 vs. 27.3 vs. 27.8 kg/m^2^, *P* < 0.001), proportion of female fetus (42.6 vs. 47.1 vs. 51.6%, *P* < 0.01), fetal birthweight (3,260 vs. 3,360 vs. 3,440 g, *P* < 0.001), and LGA (10.9 vs. 15.4 vs. 21.3%, *P* < 0.01), whereas an inverse trend was observed for SGA (12.6 vs. 8.8 vs. 6.4%, *P* < 0.01). When compared to women in the normal category of SFVB12R, the incidence of ICP and PTB were significantly higher for women in the low category (ICP: 11.2 vs. 5.9%, *P* < 0.01; PTB: 9.5 vs. 6.6%; *P* < 0.05; Table 1). There were significant differences in baseline characteristics between the SVB12 category levels, with the exception of delivery season, fetal sex, and the incidence of PE and PIH (Table 2). In addition, significant differences between the SF category levels were observed for maternal age, systolic blood pressure, gravidity, parity, gestational age, the rate of assistant production, delivery mode and season, the incidence of GDM and PE, and fetal birthweight (Table 3).

**Table 1:** Characteristics of study populations according to the categories of maternal serum folate/vitamin B12 ratio during late pregnancy.

	**Folate/vitamin B12 ratio**			***P-*** **Value**
	**Low (**<**P5;** ***N*** = **578)**	**Normal (P5–P95;** ***N*** = **10,390)**	**High (**>**P95;** ***N*** = **581)**	
Maternal age at delivery (years)	28.0 ± 4.2	28.6 ± 4.4	29.3 ± 4.9	<0.001
<35	528 (91.3%)	9,217 (88.7%)	481 (82.8%)	<0.001
≥35	50 (8.7%)	1,173 (11.3%)	100 (17.2%)	
BMI at delivery (kg/m^2^)^a^	26.6 ± 3.6	27.3 ± 3.3	27.8 ± 3.5	<0.001
<25	206 (36.1%)	2,546 (24.7%)	108 (18.7%)	<0.001
≥25	364 (63.9%)	7,744 (75.3%)	469 (81.3%)	
Systolic BP at delivery (mmHg)	121.3 ± 11.7	121.0 ± 12.1	120.8 ± 11.6	0.685
Diastolic BP at delivery (mmHg)	75.5 ± 8.1	74.5 ± 8.3	74.1 ± 8.1	0.007
**Gravidity**				
<3	424 (73.4%)	7,381 (71.0%)	405 (69.7%)	0.368
≥3	154 (26.6%)	3,009 (29.0%)	176 (30.3%)	
**Parity**				
No child	358 (61.9%)	6,245 (60.1%)	330 (56.8%)	0.180
≥1 child	220 (38.1%)	4,145 (39.9%)	251 (43.2%)	
Gestational age at delivery (week)	38.5 ± 1.6	38.7 ± 1.7	38.6 ± 1.7	0.001
Assisted reproduction	11 (1.9%)	237 (2.3%)	16 (2.8%)	0.622
**Pregnancy complication^b^**				
GDM	43 (7.4%)	874 (8.4%)	48 (8.3%)	0.711
ICP	65 (11.2%)	612 (5.9%)	34 (5.9%)	<0.001
PE	22 (3.8%)	348 (3.3%)	24 (4.1%)	0.520
PIH	18 (3.1%)	218 (2.1%)	9 (1.5%)	0.158
**Delivery mode**				
Vaginal delivery	354 (61.2%)	5,970 (57.5%)	317 (54.6%)	0.068
Cesarean section	224 (38.8%)	4,420 (42.5%)	264 (45.4%)	
**Delivery season**				
Spring	103 (17.8%)	2,303 (22.2%)	144 (24.8%)	<0.001
Summer	167 (28.9%)	2,619 (25.2%)	112 (19.3%)	
Autumn	167 (28.9%)	2,901 (27.9%)	152 (26.2%)	
Winter	141 (24.4%)	2,567 (24.7%)	173 (29.8%)	
PTB	55 (9.5%)	690 (6.6%)	45 (7.7%)	0.019
**Fetal sex**				
Female	246 (42.6%)	4,896 (47.1%)	300 (51.6%)	0.008
Male	332 (57.4%)	5,494 (52.9%)	281 (48.4%)	
Fetal birth length (cm)	49.7 ± 1.5	49.8 ± 1.4	49.9 ± 1.3	0.074
Fetal birth weight (g)	3,260 (2,930–3,550)	3,360 (3,070–3,650)	3,440 (3,150–3,710)	<0.001
SGA	73 (12.6%)	912 (8.8%)	37 (6.4%)	<0.001
AGA	442 (76.5%)	7,874 (75.8%)	420 (72.3%)	
LGA	63 (10.9%)	1,604 (15.4%)	124 (21.3%)	

Data were presented as median (IQR); mean ± SD; and N (%) for continuous variables with normal distribution; continuous variables with skewed distribution; and categorical variables, respectively.

a 112 cases missing maternal height or weight at delivery.

b 221 cases had more than one kind of complications.

IQR, interquartile range; SD, standard deviation; P, percentile; BMI, body mass index; BP, blood pressure; GDM, gestational diabetes mellitus; ICP, intrahepatic cholestasis of pregnancy; PE, pre-eclampsia; PIH, pregnancy-induced hypertension; PTB, pre-term birth; SGA/AGA/LGA, small/appropriate/large for gestational age.

**Table 2:** Characteristics of study populations according to the categories of maternal serum vitamin B12 levels during late pregnancy.

	**Vitamin B12 levels**			***P-*** **Value**
	**Low (**<**P5;** ***N*** = **561)**	**Normal (P5–P95;** ***N*** = **10,419)**	**High (**>**P95;** ***N*** = **569)**	
Maternal age at delivery (years)	27.4 ± 5.1	28.6 ± 4.4	29.3 ± 4.2	<0.001
<35	505 (90.0%)	9,227 (88.6%)	497 (86.9%)	0.253
≥35	56 (10.0%)	1,192 (11.4%)	75 (13.1%)	
BMI at delivery (kg/m^2^)^a^	28.0 ± 3.4	27.4 ± 3.4	25.9 ± 3.0	<0.001
<25	103 (18.5%)	2,541 (24.6%)	214 (37.9%)	<0.001
≥25	453 (81.5%)	7,775 (75.4%)	351 (62.1%)	
Systolic BP at delivery (mmHg)	122.1 ± 12.2	121.0 ± 12.0	119.6 ± 11.6	0.002
Diastolic BP at delivery (mmHg)	75.9 ± 8.5	74.4 ± 8.2	74.4 ± 8.3	<0.001
**Gravidity**				
<3	362 (64.5%)	7,425 (71.3%)	425 (74.3%)	0.009
≥3	199 (35.5%)	2,994 (28.7%)	147 (25.7%)	
**Parity**				
No child	278 (49.6%)	6,278 (60.3%)	379 (66.3%)	<0.001
≥1 child	283 (50.4%)	4,141 (39.7%)	193 (33.7%)	
Gestational age at delivery (week)	38.8 ± 1.7	38.7 ± 1.7	38.4 ± 1.8	<0.001
Assisted reproduction	4 (0.7%)	233 (2.2%)	27 (4.7%)	<0.001
**Pregnancy complication^b^**				
GDM	17 (3.0%)	871 (8.4%)	77 (13.5%)	<0.001
ICP	41 (7.3%)	606 (5.8%)	64 (11.2%)	<0.001
PE	28 (5.0%)	344 (3.3%)	22 (3.8%)	0.084
PIH	17 (3.0%)	221 (2.1%)	7 (1.2%)	0.108
**Delivery mode**				
Vaginal delivery	360 (64.2%)	5,988 (57.5%)	295 (51.6%)	<0.001
Cesarean section	201 (35.8%)	4,431 (42.5%)	277 (48.4%)	
**Delivery season**				
Spring	152 (27.1%)	2,284 (21.9%)	115 (20.1%)	0.053
Summer	118 (21.0%)	2,626 (25.2%)	155 (27.1%)	
Autumn	157 (28.0%)	2,906 (27.9%)	158 (27.6%)	
Winter	134 (23.9%)	2,603 (25.0%)	144 (25.2%)	
PTB	41 (7.3%)	694 (6.7%)	55 (9.6%)	0.022
**Fetal sex**				
Female	287 (51.2%)	4,896 (47.0%)	261 (45.6%)	0.119
Male	274 (48.8%)	5,523 (53.0%)	311 (54.4%)	
Fetal birth length (cm)	49.9 ± 1.3	49.8 ± 1.4	49.6 ± 1.9	0.001
Fetal birth weight (g)	3,400 (3,050–3,710)	3,360 (3,080–3,660)	3,250 (2,950–3,513)	<0.001
SGA	52 (9.3%)	898 (8.6%)	72 (12.6%)	<0.001
AGA	409 (72.9%)	7,888 (75.7%)	442 (77.3%)	
LGA	100 (17.8%)	1,633 (15.7%)	58 (10.1%)	

Data were presented as median (IQR); mean ± SD; and N (%) for continuous variables with normal distribution; continuous variables with skewed distribution; and categorical variables, respectively.

a 112 cases missing maternal height or weight at delivery.

b 221 cases had more than one kind of complications.

IQR, interquartile range; SD, standard deviation; P, percentile; BMI, body mass index; BP, blood pressure; GDM, gestational diabetes mellitus; ICP, intrahepatic cholestasis of pregnancy; PE, pre-eclampsia; PIH, pregnancy-induced hypertension; PTB, pre-term birth; SGA/AGA/LGA, small/appropriate/large for gestational age.

**Table 3:** Characteristics of study populations according to the categories of maternal serum folate levels during late pregnancy.

	**Folate levels (**μ**g/L)**			***P-*** **Value**
	**Low (**<**P5;** ***N*** = **574)**	**Normal (P5–P95;** ***N*** = **10,073)**	**High (**>**P95;** ***N*** = **902)^c^**	
Maternal age at delivery (years)	26.7 ± 4.5	28.6 ± 4.4	29.8 ± 4.9	<0.001
<35	546 (95.1%)	8,928 (88.6%)	753 (83.4%)	<0.001
≥35	28 (4.9%)	1,145 (11.4%)	150 (16.6%)	
BMI at delivery (kg/m^2^)^a^	27.5 ± 3.9	27.3 ± 3.3	27.1 ± 3.3	0.062
<25	150 (26.6%)	2,472 (24.8%)	236 (26.3%)	0.382
≥25	413 (73.4%)	7,504 (75.2%)	660 (73.7%)	
Systolic BP at delivery (mmHg)	122.0 ± 12.4	121.0 ± 12.0	120.1 ± 12.0	0.009
Diastolic BP at delivery (mmHg)	75.4 ± 8.4	74.5 ± 8.2	74.2 ± 8.4	0.061
**Gravidity**				
<3	404 (70.4%)	7,124 (70.7%)	682 (75.5%)	0.009
≥3	170 (29.6%)	2,949 (29.3%)	221 (24.5%)	
**Parity**				
No child	318 (55.4%)	6,018 (59.7%)	597 (66.1%)	<0.001
≥1 child	256 (44.6%)	4,055 (40.3%)	306 (33.9%)	
Gestational age at delivery (week)	38.6 ± 1.8	38.7 ± 1.7	38.5 ± 1.6	0.005
Assisted reproduction	5 (0.9%)	212 (2.1%)	47 (5.2%)	<0.001
**Pregnancy complication^b^**				
GDM	13 (2.3%)	837 (8.3%)	115 (12.7%)	<0.001
ICP	47 (8.2%)	616 (6.1%)	48 (5.3%)	0.073
PE	34 (5.9%)	325 (3.2%)	35 (3.9%)	0.002
PIH	13 (2.3%)	213 (2.1%)	19 (2.1%)	0.970
**Delivery mode**				
Vaginal delivery	368 (64.1%)	5,845 (58.0%)	428 (47.4%)	<0.001
Cesarean section	206 (35.9%)	4,228 (42.0%)	475 (52.6%)	
**Delivery season**				
Spring	109 (19.0%)	2,298 (22.8%)	143 (15.8%)	<0.001
Summer	161 (28.0%)	2,536 (25.2%)	201 (22.3%)	
Autumn	173 (30.1%)	2,738 (27.2%)	309 (34.2%)	
Winter	131 (22.8%)	2,501 (24.8%)	250 (27.7%)	
PTB	58 (10.1%)	668 (6.6%)	65 (7.2%)	0.005
**Fetal sex**				
Female	275 (47.9%)	4,710 (46.8%)	458 (50.7%)	0.068
Male	299 (52.1%)	5,363 (53.2%)	445 (49.3%)	
Fetal birth length (cm)	49.7 ± 1.6	49.8 ± 1.4	49.9 ± 1.5	0.060
Fetal birth weight (g)	3,330 (3,000–3,610)	3,360 (3,070–3,650)	3,340 (3,070–3,625)	0.008
SGA	66 (11.5%)	886 (8.8%)	70 (7.8%)	0.134
AGA	427 (74.4%)	7,614 (75.6%)	696 (77.1%)	
LGA	81 (14.1%)	1,573 (15.6%)	137 (15.2%)	

Data were presented as median (IQR); mean ± SD; and N (%) for continuous variables with normal distribution; continuous variables with skewed distribution; and categorical variables; respectively.

a 112 cases missing maternal height or weight at delivery.

b 221 cases had more than one kind of complications.

c 896 cases had a same folate level of 24.2 μg/L.

IQR, interquartile range; SD, standard deviation; P, percentile; BMI, body mass index; BP, blood pressure; GDM, gestational diabetes mellitus; ICP, intrahepatic cholestasis of pregnancy; PE, pre-eclampsia; PIH, pregnancy-induced hypertension; PTB, pre-term birth; SGA/AGA/LGA, small/appropriate/large for gestational age.

### Maternal SF, SVB12, SFVB12R, and fetal development

Compare with those with AGA neonates, the mothers with SGA had lower levels of SF and SFVB12R (median for SF: 9.07 vs. 9.88 μg/L, *P* < 0.01; for SFVB12R: 47.16 vs. 50.63, *P* < 0.01), while the mothers with LGA had a lower SVB12 level and a higher SFVB12R level (median for SVB12: 195 vs. 201 ng/L, *P* < 0.01; for SFVB12R: 52.68 vs. 50.63, *P* < 0.05). The levels of SF and SFVB12R in mothers with PTB were significantly lower compared with those in FTB (median for SF: 8.98 vs. 9.88 μg/L, *P* < 0.01; for SFVB12R: 46.46 vs. 50.83, *P* < 0.01; Table 4). Compared to women with normal SFVB12R, mean birthweight in women with high SFVB12R significantly increased by 60.99 g (95% CI: 29.52, 92.45), while mean birthweight in women with low SFVB12R decreased by 43.81 g (95% CI: −75.62, −12.00), respectively (all *P* < 0.01). In addition, high SVB12 significantly decreased gestational age and birthweight by 0.20 week (95% CI: −0.34, −0.06) and 61.87 g (95% CI: −94.26, −29.47), respectively (all *P* < 0.01; Table 5).

**Table 4:** The distribution of maternal serum levels of folate, vitamin B12, and their ratio on admission for labor according to birth outcomes.

	***N***	**P 5**	**P 10**	**P 25**	**Median**	**P 75**	**P 90**	**P 95**	***P*** **-Value**
SFVB12R									
All women	11,549	20.47	24.56	34.17	50.51	75.68	104.00	124.74	
AGA	8,736	20.43	24.66	34.29	50.63	75.59	103.84	123.47	
SGA	1,022	18.15	22.56	31.32	47.16	68.88	91.53	112.63	<0.001^a^
LGA	1,791	21.81	25.47	35.66	52.68	79.36	110.95	135.42	0.011^a^
FTB	10,759	20.59	24.75	34.35	50.83	75.96	104.19	124.60	
PTB	790	18.36	22.11	30.76	46.46	70.79	102.23	127.80	0.001^b^
SF (μg/L)									
All women	11,549	4.25	4.96	6.59	9.81	16.31	23.30	24.20	
AGA	8,736	4.27	4.96	6.59	9.88	16.31	23.30	24.20	
SGA	1,022	4.07	4.73	6.25	9.07	15.48	23.27	24.20	0.007^a^
LGA	1,791	4.35	5.07	6.76	9.96	16.86	23.30	24.20	0.511^a^
FTB	10,759	4.29	5.01	6.64	9.88	16.33	23.30	24.20	
PTB	790	3.85	4.49	5.98	8.98	15.78	23.30	24.20	0.001^b^
SVB12 (ng/L)									
All women	11,549	106.00	120.00	154.00	200.00	260.00	333.90	390.00	
AGA	8,736	106.00	120.24	154.00	201.00	261.00	336.00	391.10	
SGA	1,022	105.05	123.00	157.00	208.00	269.00	352.90	420.95	0.086^a^
LGA	1,791	104.00	118.00	152.00	195.00	254.00	317.00	358.00	0.007^a^
FTB	10,759	106.00	120.00	154.00	200.00	260.00	333.00	387.00	
PTB	790	105.00	118.00	148.00	197.50	264.00	346.30	436.75	0.514^b^

a Compared with FTB group.

b Compared with AGA group.

P, percentile; SFVB12R, serum folate to vitamin B12 ratio; SGA/AGA/LGA, small/appropriate/large for gestational age; FTB, full-term birth; PTB, pre-term birth; SF, serum folate; SVB12, serum vitamin B12.

**Table 5:** β (95% CI) for fetal development associated with categories of maternal SF, SVB12 levels, and their ratio.

	**Gestational age (weeks)**		**Birth weight (g)**		**Birth length (cm)**	
	β **(95% CI)**	***P-*** **Value**	β **(95% CI)**	***P-*** **Value**	β **(95% CI)**	***P-*** **Value**
**Unadjusted**						
**SFVB12R**						
P5–P95	Ref.		Ref.		Ref.	
>P95	−0.13 (−0.27, 0.01)	0.062	59.73 (18.38, 101.08)	0.005	0.04 (−0.07, 0.16)	0.463
< P5	−0.23 (−0.37, −0.09)	0.002	−110.37 (−151.84, −68.90)	<0.001	−0.13 (−0.25, −0.01)	0.034
**SF**						
P5–P95	Ref.		Ref.		Ref.	
>P95	−0.17 (−0.28, −0.05)	0.004	−24.96 (−58.62, 8.71)	0.146	−0.02 (−0.12, 0.08)	0.690
< P5	−0.13 (−0.27, 0.01)	0.079	−60.84 (−102.65, −19.04)	0.004	−0.16 (−0.28, −0.04)	0.010
**SVB12**						
P5–P95	Ref.		Ref.		Ref.	
>P95	−0.26 (−0.40, −0.12)	<0.001	−138.61 (−180.25, −96.97)	<0.001	−0.22 (−0.34, −0.10)	<0.001
< P5	0.10 (−0.05, 0.24)	0.184	22.87 (−19.19, 64.92)	0.287	0.03 (−0.09, 0.15)	0.660
**Adjusted**						
**SFVB12R^a^**						
P5–P95	Ref.		Ref.		Ref.	
>P95	−0.14 (−0.27, 0.00)	0.045	60.99 (29.52, 92.45)	<0.001	0.07 (−0.02, 0.16)	0.118
< P5	−0.18 (−0.32, −0.05)	0.008	−43.81 (−75.62, −12.00)	0.007	0.02 (−0.07, 0.12)	0.594
**SF^b^**						
P5–P95	Ref.		Ref.		Ref.	
>P95	−0.12 (−0.23, −0.01)	0.037	19.08 (−7.03, 45.18)	0.152	0.06 (−0.01, 0.14)	0.090
< P5	−0.07 (−0.21, 0.07)	0.301	−29.25 (−61.74, 3.25)	0.078	−0.03 (−0.12, 0.06)	0.508
**SVB12^c^**						
P5–P95	Ref.		Ref.		Ref.	
>P95	−0.20 (−0.34, −0.06)	0.004	−61.87 (−94.26, −29.47)	<0.001	−0.08 (−0.17, 0.01)	0.093
< P5	0.12 (−0.01, 0.26)	0.077	6.33 (−26.18, 38.84)	0.703	0.00 (−0.09, 0.09)	0.983

a β-values for gestational age were adjusted for maternal age, BMI, gravidity, parity, blood pressure, GDM, ICP, PE, PIH, and fetal sex; β-values for birthweight and birth length were additionally adjusted for gestational age.

b Additionally adjusted for SVB12 levels.

c Additionally adjusted for SF levels.

OR, odds ratio; CI, confidence interval; SFVB12R, serum folate to vitamin B12 ratio; P; percentile; SF, serum folate, SVB12, serum vitamin B12; GDM, gestational diabetes mellitus; ICP, intrahepatic cholestasis of pregnancy; PE, pre–eclampsia; PIH, pregnancy induced hypertension; BMI, body mass index.

### Maternal SF, SVB12, SFVB12R, and pregnancy outcomes

**Figure 2:**
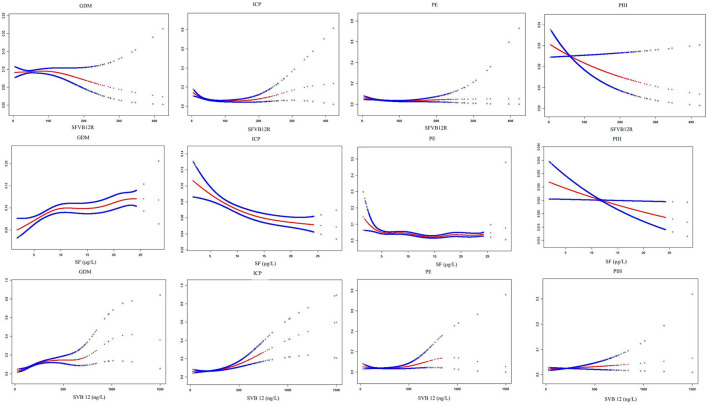
Smooth curve fitting analysis of SF, SVB12, and SFVB12R with the risk of pregnancy complications. For SFVB12R vs. pregnancy complications, adjusting for maternal age, BMI, gravidity, parity; for SF vs. pregnancy complications, additionally adjusting for SVB12; for SVB12 vs. pregnancy complications, additionally adjusting for SF. SFVB12R, serum folate to vitamin B12 ratio; SF, serum folate, SVB12, serum vitamin B12; GDM, gestational diabetes mellitus; ICP, intrahepatic cholestasis of pregnancy; PE, pre-eclampsia; PIH, pregnancy induced hypertension.

**Figure 3:**
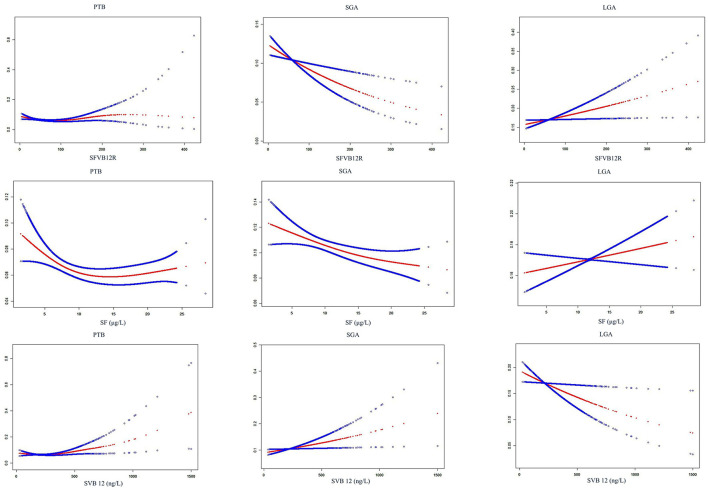
Smooth curve fitting analysis of SF, SVB12, and SFVB12R with the risk of adverse birth outcomes. For SFVB12R vs. PTB, adjusting for maternal age, BMI, gravidity, parity, blood pressure, GDM, ICP, PE, PIH, and fetal sex; for SFVB12R vs. SGA/LGA, additionally adjusting for gestational age; for SF vs. PTB, additionally adjusting for SVB12; for SF vs. SGA/LGA, additionally adjusting for SVB12; for SVB12 vs. PTB, additionally adjusting for SF; for SVB12 vs. SGA/LGA, additionally adjusting for SF. SF, serum folate; SVB12, serum vitamin B12; SFVB12R, serum folate to vitamin B12 ratio; GDM, gestational diabetes mellitus; ICP, intrahepatic cholestasis of pregnancy; PE, pre-eclampsia; PIH, pregnancy-induced hypertension; PTB, pre-term birth; SGA/LGA, small/large for gestational age.

Associations of maternal SF, SVB12, and SFVB12R with risk for adverse pregnancy outcomes in unadjusted and adjusted logistic regression models were shown in Tables 6, 7. Low SFVB12R was associated with increased ICP and PIH, and with decreased LGA; the adjusted ORs (95% CI) were 2.03 (1.54–2.67), 1.81 (1.09–3.00), and 0.75 (0.56–1.00), respectively. High SFVB12R was associated with increased LGA; the adjusted OR (95% CI) was 1.32 (1.06–1.65). High SVB12 was associated with increased GDM, ICP, PE, and PTB, and with decreased LGA; the adjusted ORs (95% CI) were 1.62 (1.24–2.11), 2.22 (1.67–2.96), 1.69 (1.04–2.74), and 0.70 (0.52–0.94), respectively. In addition, low SF was associated with increased ICP and PE, and with decreased GDM; the adjusted ORs (95% CI) were 1.58 (1.15–2.17), 1.89 (1.28–2.81), and 0.40 (0.25–0.69), respectively. The smooth curve fitting analysis revealed that maternal SF, SVB12, and SVB12R levels were associated with risks of pregnancy outcomes (GDM/ICP/PE/PTB) in a nonlinear fashion (Figures 2, 3).

**Table 6:** ORs and 95% CIs for different pregnancy complications with categories of maternal SF, SVB12 levels, and their ratio.

	**GDM**		**ICP**		**PE**		**PIH**	
	**OR (95% CI)**	***P-*** **Value**	**OR (95% CI)**	***P-*** **Value**	**OR (95% CI)**	***P-*** **Value**	**OR (95% CI)**	***P-*** **Value**
**Unadjusted**								
**SFVB12R**								
P5–P95	Ref.		Ref.		Ref.		Ref.	
>P95	0.98 (0.73, 1.34)	0.920	1.00 (0.70, 1.42)	0.982	1.24 (0.81, 1.89)	0.327	0.74 (0.38, 1.45)	0.381
< P5	0.94 (0.68, 1.30)	0.712	2.03 (1.55, 2.67)	<0.001	1.21 (0.78, 1.88)	0.398	1.58 (0.97, 2.58)	0.067
**SF**								
P5–P95	Ref.		Ref.		Ref.		Ref.	
>P95	1.58 (1.28, 1.95)	<0.001	0.90 (0.66, 1.22)	0.488	1.24 (0.87, 1.77)	0.235	1.03 (0.64, 1.65)	0.909
< P5	0.27 (0.16, 0.47)	<0.001	1.33 (0.97, 1.81)	0.074	1.82 (1.26, 2.62)	0.001	1.06 (0.60, 1.87)	0.836
**SVB12**								
P5–P95	Ref.		Ref.		Ref.		Ref.	
>P95	1.77 (1.37, 2.27)	<0.001	2.11 (1.60, 2.78)	<0.001	1.28 (0.82, 1.99)	0.278	0.63 (0.30, 1.35)	0.236
< P5	0.36 (0.22, 0.59)	<0.001	1.25 (0.90, 1.74)	0.182	1.51 (1.01, 2.24)	0.043	1.42 (0.86, 2.35)	0.168
**Adjusted**								
**SFVB12R^a^**								
P5–P95	Ref.		Ref.		Ref.		Ref.	
>P95	0.82 (0.60, 1.13)	0.230	0.99 (0.69, 1.42)	0.962	0.99 (0.63, 1.54)	0.948	0.68 (0.34, 1.34)	0.261
< P5	1.05 (0.75, 1.47)	0.768	2.03 (1.54, 2.67)	<0.001	1.29 (0.80, 2.07)	0.303	1.81 (1.09, 3.00)	0.021
**SF^b^**								
P5–P95	Ref.		Ref.		Ref.		Ref.	
>P95	1.23 (0.99, 1.53)	0.065	0.76 (0.55, 1.03)	0.077	1.14 (0.79, 1.67)	0.482	1.05 (0.65, 1.72)	0.835
< P5	0.40 (0.23, 0.70)	0.001	1.58 (1.15, 2.17)	0.005	1.89 (1.28, 2.81)	0.002	1.11 (0.62, 2.00)	0.718
**SVB12^c^**								
P5–P95	Ref.		Ref.		Ref.		Ref.	
>P95	1.62 (1.24, 2.11)	<0.001	2.22 (1.67, 2.96)	<0.001	1.69 (1.04, 2.74)	0.033	0.88 (0.40, 1.90)	0.740
< P5	0.41 (0.25, 0.69)	0.001	1.23 (0.88, 1.72)	0.227	1.22 (0.80, 1.87)	0.351	1.32 (0.79, 2.21)	0.296

a OR values were adjusted for maternal age, BMI, gravidity, parity.

b Additionally adjusted for SVB12 levels.

c Additionally adjusted for SF levels.

OR, odds ratio; CI, confidence interval; SFVB12R, serum folate to vitamin B12 ratio; P; percentile; SF, serum folate, SVB12, serum vitamin B12; GDM, gestational diabetes mellitus; ICP, intrahepatic cholestasis of pregnancy; PE, pre-eclampsia; PIH, pregnancy induced hypertension; BMI, body mass index.

**Table 7:** ORs and 95% CIs for adverse birth outcomes with maternal SF, SVB12 levels, and their ratio.

	**PTB**		**SGA**		**LGA**	
	**OR (95% CI)**	***P-*** **Value**	**OR (95% CI)**	***P-*** **Value**	**OR (95% CI)**	***P-*** **Value**
**Unadjusted**						
**SFVB12R**						
P5–P95	Ref.		Ref.		Ref.	
>P95	1.18 (0.86, 1.62)	0.301	0.76 (0.54, 1.07)	0.118	1.45 (1.18, 1.78)	<0.001
< P5	1.48 (1.11, 1.97)	0.008	1.43 (1.10, 1.84)	0.007	0.70 (0.53, 0.92)	0.009
**SF**						
P5–P95	Ref.		Ref.		Ref.	
>P95	1.09 (0.84, 1.42)	0.514	0.86 (0.67, 1.12)	0.263	0.95 (0.79, 1.15)	0.62
< P5	1.58 (1.19, 2.10)	0.001	1.33 (1.02, 1.74)	0.038	0.92 (0.72, 1.17)	0.493
**SVB12**						
P5–P95	Ref.		Ref.		Ref.	
>P95	1.49 (1.12, 1.99)	0.007	1.43 (1.10, 1.85)	0.007	0.63 (0.48, 0.84)	0.001
< P5	1.10 (0.80, 1.53)	0.55	1.12 (0.83, 1.50)	0.466	1.18 (0.94, 1.48)	0.147
**Adjusted**						
**SFVB12R^a^**						
P5–P95	Ref.		Ref.		Ref.	
>P95	1.28 (0.92, 1.77)	0.138	0.82 (0.58, 1.16)	0.26	1.32 (1.06, 1.65)	0.013
< P5	1.33 (0.98, 1.80)	0.069	1.24 (0.95, 1.63)	0.112	0.75 (0.56, 1.00)	0.049
**SF^b^**						
P5–P95	Ref.		Ref.		Ref.	
>P95	1.07 (0.81, 1.42)	0.616	0.81 (0.62, 1.06)	0.118	0.99 (0.81, 1.22)	0.953
< P5	1.27 (0.92, 1.74)	0.141	1.20 (0.90, 1.61)	0.211	0.91 (0.69, 1.18)	0.458
**SVB12^c^**						
P5–P95	Ref.		Ref.		Ref.	
>P95	1.46 (1.07, 1.99)	0.016	1.31 (1.00, 1.73)	0.053	0.70 (0.52, 0.94)	0.017
< P5	0.97 (0.69, 1.38)	0.877	1.10 (0.80, 1.50)	0.553	1.19 (0.93, 1.51)	0.159

a OR values for gestational age were adjusted for maternal age, BMI, gravidity, parity, blood pressure, GDM, ICP, PE, PIH, and fetal sex; OR values for SGA and birth LGA were additionally adjusted for gestational age.

b Additionally adjusted for SVB12 levels.

c Additionally adjusted for SF levels.

OR, odds ratio; CI, confidence interval; PTB, pre-term birth; SGA/LGA, small/large for gestational age; SFVB12R, serum folate to vitamin B12 ratio; P; percentile; SF, serum folate, SVB12, serum vitamin B12; GDM, gestational diabetes mellitus; ICP, intrahepatic cholestasis of pregnancy; PE, pre-eclampsia; PIH, pregnancy induced hypertension; BMI, body mass index.

## Discussion

In this observational study, we comprehensively described the maternal profile of SF, SVB12, and their ratio in accordance with perinatal outcomes in the Chinese population and investigated the associations of maternal imbalance between folate and vitamin B12 with pregnancy complications and perinatal outcomes. Our retrospective study showed three important findings. First, it is the first report from China to suggest that higher SFVB12R increase the birthweight and is positively associated with the risk of LGA; while lower SFVB12R decrease the birthweight and is negatively associated with the risk of LGA; Second, we first reported that lower SFVB12R is associated with higher risks of ICP and PIH. Third, we conformed that abnormal maternal levels of SF and SVB12 are associated with the risks of pregnancy complications.

Folate and Vitamin B12 participate in the one-carbon metabolism cycle and are essential for the regulation of fetal growth (17). Insufficient levels of folate or vitamin B12 during pregnancy are associated with an increased risk of neural tube defects, poorer cognitive function, spontaneous abortion, stillbirth, fetal growth restriction, preterm delivery, and low birthweight (18–26). In the present study, we showed that lower SF levels (<5th percentile) reduced the birthweight and were positively related to SGA risk in unadjusted models, which were consistent with the previous cohort study by Baker et al. who reported that low folate status was associated with increased risk of SGA births (18). In our study, we found that the association between lower SF and high SGA did not show statistical significance after adjusting for SVB12 levels and other confounding factors. Furthermore, we showed no significant association of low vitamin B12 levels (<5th percentile) with birthweight and SGA risk in the unadjusted and adjusted analysis, which is in agreement with the population-based cohort study (27). In addition, inadequate maternal folate or vitamin B12 status has been linked to a high risk of PE or GDM (28, 29). In this observational study, we confirmed that lower SF levels were associated with a higher incidence of PE and ICP. To our best knowledge, this is the first study that correlated lower SF levels with higher ICP prevalence. In addition, we found that lower SVB12 levels decreased the risk of GDM (adjusted-OR: 0.41), while higher SVB12 levels increased the risk (adjusted-OR: 1.62). These results seem to be in accord with the recent study in China, in which vitamin B12 levels were positively associated with GDM risk (OR: 1.14 per 100 pg/mL) (11).

There is increasing recognition of the adverse outcomes caused by high levels and/or imbalance of folate and vitamin B12 with the widespread use of prenatal folate supplements. Cumulative evidence shows that high maternal folate levels and/or imbalance of folate and vitamin B12, represented by high folate and low vitamin B12 or high SFVB12R, increase GDM risk during pregnancy (9–12, 30). However, a Nurses' Health Study from the USA reported that high supplements of folate before pregnancy significantly decreased GDM risk (31). Our study indicated that higher SF levels, but not SFVB12R, were associated with increased risk of GDM in unadjusted models (OR: 1.58); however, after adjusting for available confounders, the *P*-value for the association changed from <0.001 to 0.065. Furthermore, we found no significant association between high SFVB12R and GDM in unadjusted and adjusted analysis. Similarly, a recent cohort study from China reported that the folate/vitamin B12 ratio from red blood cells was not associated with GDM risk (11). In an observational study from India, 49 full-term pregnant women indicated that an increased ratio of folate to vitamin B12 decreased neonatal birthweight (13). Another observational study on 492 Indian pregnant women suggested that high folate and low vitamin B12 intakes during pregnancy were associated with an increased risk of SGA (32). However, results from a recent cohort study on 2,632 Dutch pregnant women indicated that high folate and low vitamin B12 intakes in late pregnancy were not associated with birthweight (33). Conversely, our study indicated that high SFVB12R increased the birthweight (β: 60.99 g) and was positively associated with LGA risk (OR: 1.32). In addition, we observed a positive correlation between the levels of SF and SVB12 in our study population (*r* = 0.35; *P* < 0.001; data not shown). It is thus likely that the participants in this study took multivitamins in which proportions of folate and vitamin B12 were fixed. However, excess vitamin B12 status (or intake) during late pregnancy might be a concern. We first reported that higher SVB12 levels were associated with increased risks of ICP (OR: 2.22) and PE (OR: 1.69). In addition, high SVB12 decreased the birthweight (β: −138.61 g) and increased the risk of SGA by 1.43-fold in the crude logistic regression model. The *P*-value for the association between SVB12 and SGA risk changed from 0.007 to 0.053 after adjusting for the confounders. Case-control and cohort studies examining the association between maternal folate, vitamin B12 level, and their ratio, and adverse pregnancy outcomes have not reported consistent conclusions. Although the reasons for this discrepancy are unclear, it could be due to the differences in the gestational weeks when measurements of folate and vitamin B12 were taken between the studies. Longitudinal studies demonstrated that folate and vitamin B12 levels decrease significantly with the progress of pregnancy and reached the lowest point at delivery (34, 35). The discrepancies might be because of differences in study population frequencies of the MTHFR 677C>T polymorphism that could play a role in fetal growth and placental abruption (36, 37). Additionally, different cut-off criteria defining abnormal folate, vitamin B12 levels, and their ratio could contribute to this discrepancy, including tertiles, quartiles, different percentiles, and cut-off values.

The mechanisms by which abnormal maternal folate and vitamin B12 levels confer an increased risk of adverse pregnancy outcomes have not been well understood. Evidence from animal models revealed that maternal folate deficiency inhibited mTOR signaling, downregulates placental amino acid transporters, and led to fetal growth restriction in mice; B12 supplementation significantly upregulated placental miR-16 and miR-21 related to fetal growth and improved the birthweight of rats (38, 39). In human placental studies, low folate status has been shown to impact trophoblast viability and may alter the transport of nutrients to the fetus by mTOR folate sensing, and alter the miRNA expression and contribute to placental dysfunction underpinning common pregnancy disorders such as pre-eclampsia and fetal growth restriction (40, 41). In addition, both low and high folate levels had an adverse impact on placental development and function; B12 treatment may effectively neutralize the effect of excessive folic acid *in vitro* (42, 43). Moreover, an *in vivo* rodent study elucidated altered dietary ratios of folate and B12 could have more severe effects than individual deficiencies on the expression of transporters, related miRNAs, and DNA methylation in C57BL/6 mice, which play an important role in fetal development (44). Therefore, an imbalance between folate and vitamin B12 during pregnancy could theoretically cause adverse pregnancy outcomes through placental mechanisms (17). Our findings provide new insights for further study on the effect of maternal folate and vitamin B12 imbalance on birthweight alteration.

The strengths of this cohort study included the fact that its large sample size of 11,549 pregnant women with various levels of folate and vitamin B12 ensuring sufficient and reliable statistical power to determine the associations of an imbalance status between folate and vitamin B12 with adverse pregnancy outcomes by categories of SFVB12R. Additionally, folate and vitamin B12 levels were investigated in a single laboratory using the same instruments and settings minimizing test-induced variability. Finally, some available confounding factors were adjusted in the statistical analysis. However, several limitations of our retrospective study should be mentioned. First, detailed information on maternal socioeconomic status, history of pregnancy complications, dietary intake of vitamin B12 and folate, and medication for B vitamins deficiency was lacking, which may affect the statistical analysis. However, serum levels more accurately reflect the storage of folate and vitamin B12 in the body rather than diet assessment by self-report. Second, the present study was an observational cohort study, and it did not prove the levels of other metabolic markers of B vitamins such as methylmalonic acid, although methylmalonic acid levels are more sensitive and specific indicators of folate and vitamin B12 status. Third, due to the lack of folate and vitamin B12 before pregnancy and in early and mid pregnancy, our study did not investigate persistent effects caused by an imbalance of the B vitamins from early pregnancy to late pregnancy. Therefore, a prospective cohort study with a large population and improved design would be beneficial to further investigate the fluctuation of B vitamins imbalance in a longer observational period of reproductive-age women to determine its associations with adverse pregnancy outcomes.

In conclusion, our observational study displayed the maternal profile of SF, SVB12, and their ratio at hospital admission for delivery and suggested that an imbalance between folate and vitamin B12, represented by a higher or lower SFVB12R is significantly associated with adverse pregnancy outcomes (ICP/PIH/LGA).

## Data availability statement

The raw data supporting the conclusions of this article will be made available by the authors, without undue reservation.

## Ethics statement

The studies involving human participants were reviewed and approved by Changzhou Maternal and Child Health Care Hospital Ethics Committee. The patients/participants provided their written informed consent to participate in this study.

## Author contributions

BY and BZ conceived and designed this study. XY wrote the manuscript. XH and WZ collected the data. HW interpreted the reports. All authors listed have made a substantial, direct, and intellectual contribution to the work and approved it for publication.

## Funding

This work was supported by the Jiangsu Maternal and Child Health Research Projects (F201842), the Science and technology project for young talents of Changzhou Health Commission (QN202048), and the General project of Jiangsu Provincial Health Commission (M2021079).

## Conflict of interest

The authors declare that the research was conducted in the absence of any commercial or financial relationships that could be construed as a potential conflict of interest.

## Publisher's note

All claims expressed in this article are solely those of the authors and do not necessarily represent those of their affiliated organizations, or those of the publisher, the editors and the reviewers. Any product that may be evaluated in this article, or claim that may be made by its manufacturer, is not guaranteed or endorsed by the publisher.

## Abbreviations

SF: serum ferritin
SVB12: serum vitamin B12
SFVB12R: serum folate to vitamin B12 ratio
SGA/AGA/LGA: small/appropriate/large for gestational age
FTB: full-term birth
PTB: pre-term birth
PE: pre-eclampsia
GDM: gestational diabetes mellitus
ICP: intrahepatic cholestasis of pregnancy
PIH: pregnancy-induced hypertension
BMI: body mass index
OR: odds ratio
CI: confidence interval.
