# Supplementary material for: Association of folate and vitamin B12 imbalance with adverse pregnancy outcomes among 11,549 pregnant women: An observational cohort study
Source: Front Nutr. 2022 Jul 25;9:947118. doi: 10.3389/fnut.2022.947118 (PMC9358651; doi:10.3389/fnut.2022.947118)
Supplement: Supplementary file 1 [file Data_Sheet_1.doc]

| **Table S1** Characteristics of study populations according to the categories of maternal serum folate/vitamin B12 ratio during late pregnancy. | | | | |
| --- | --- | --- | --- | --- |
|  | Folate/vitamin B12 ratio | | | *P* value |
|  | Low (<P10; N=1155) | Normal (P10-P90; N=9239) | High (>P90; N=1155) |
| Maternal age at delivery (years) | 28.0 ± 4.1 | 28.6 ± 4.4 | 29.2 ± 4.7 | <0.001 |
| <35 | 1061 (91.9%) | 8192 (88.7%) | 973 (84.2%) | <0.001 |
| ≥35 | 94 (8.1%) | 1047 (11.3%) | 182 (15.8%) |
| BMI at delivery (kg/m^2^) ^a^ | 26.9 ± 3.6 | 27.3 ± 3.3 | 27.7 ± 3.4 | <0.001 |
| <25 | 369 (32.5%) | 2262 (24.7%) | 227 (19.8%) | <0.001 |
| ≥25 | 768 (67.5%) | 6888 (75.3%) | 920 (80.2%) |
| Systolic BP at delivery (mmHg) | 121.1 ± 12.0 | 121.0 ± 12.1 | 120.7 ± 11.6 | 0.738 |
| Diastolic BP at delivery(mmHg) | 75.1 ± 8.2 | 74.5 ± 8.3 | 74.2 ± 8.1 | 0.037 |
| Gravidity |  | | | |
| <3 | 840 (72.7%) | 6558 (71.0%) | 812 (70.3%) | 0.385 |
| ≥3 | 315 (27.3%) | 2681 (29.0%) | 343 (29.7%) |
| Parity |  | | | |
| No child | 701 (60.7%) | 5537 (59.9%) | 695 (60.2%) | 0.878 |
| ≥1 child | 454 (39.3%) | 3702 (40.1%) | 460 (39.8%) |
| Gestational age at delivery (week) | 38.5 ± 1.7 | 38.7 ± 1.7 | 38.7 ± 1.5 | 0.001 |
| Assisted reproduction | 18 (1.6%) | 218 (2.4%) | 28 (2.4%) | 0.217 |
| Pregnancy complication ^b^ |  |  |  |  |
| GDM | 86 (7.4%) | 775 (8.4%) | 104 (9.0%) | 0.388 |
| ICP | 116 (10.0%) | 535 (5.8%) | 60 (5.2%) | <0.001 |
| PE | 46 (4.0%) | 310 (3.4%) | 38 (3.3%) | 0.526 |
| PIH | 32 (2.8%) | 191 (2.1%) | 22 (1.9%) | 0.255 |
| Delivery mode |  | | | |
| Vaginal delivery | 681 (59.0%) | 5321 (57.6%) | 639 (55.3%) | 0.194 |
| Cesarean section | 474 (41.0%) | 3918 (42.4%) | 516 (44.7%) |
| Delivery season |  |  |  |  |
| Spring | 224 (19.4%) | 2060 (22.3%) | 266 (23.0%) | <0.001 |
| Summer | 329 (28.5%) | 2348 (25.4%) | 221 (19.1%) |
| Autumn | 339 (29.4%) | 2545 (27.5%) | 336 (29.1%) |
| Winter | 263 (22.8%) | 2286 (24.7%) | 332 (28.7%) |
| PTB | 110 (9.5%) | 609 (6.6%) | 71 (6.1%) | <0.001 |
| Fetal sex |  |  |  |  |
| Female | 500 (43.3%) | 4360 (47.2%) | 582 (50.4%) | 0.003 |
| Male | 655 (56.7%) | 4879 (52.8%) | 573 (49.6%) |
| Fetal birth length (cm) | 49.7 ± 1.6 | 49.8 ± 1.4 | 49.9 ± 1.1 | 0.005 |
| Fetal birth weight (g) | 3270 (3000–3570) | 3360 (3070–3650) | 3400 (3130–3700) | <0.001 |
| SGA | 135 (11.7%) | 815 (8.8%) | 72 (6.2%) | <0.001 |
| AGA | 862 (74.6%) | 7003 (75.8%) | 871 (75.4%) |
| LGA | 158 (13.7%) | 1421 (15.4%) | 212 (18.4%) |
| **Notes:** Data were presented as median (IQR); mean ±SD and N (%) for continuous variables with normal distribution; continuous variables with skewed distribution; and categorical variables; respectively.  ^a^ 112 cases missing maternal height or weight at delivery.  ^b^ 221 cases had more than one kind of complications.  **Abbreviations:** IQR; interquartile range; SD; standard deviation; P; percentile; BMI; body mass index; BP; blood pressure; GDM, gestational diabetes mellitus; ICP, intrahepatic cholestasis of pregnancy; PE, preeclampsia; PIH, pregnancy-induced hypertension; PTB, pre-term birth; SGA/AGA/LGA, small/appropriate/large for gestational age | | | | |

| **Table S2** Characteristics of study populations according to the categories of maternal serum vitamin B12 levels during late pregnancy | | | | |
| --- | --- | --- | --- | --- |
|  | Vitamin B12 levels | | | *P* value |
|  | Low (<P10; N=1122) | Normal (P10-P90; N=9274) | High (>P90; N=1156) |
| Maternal age at delivery (years) | 27.6 ± 4.9 | 28.6 ± 4.4 | 29.3 ± 4.2 | <0.001 |
| <35 | 1013 (90.3%) | 8214 (88.6%) | 1002 (86.7%) | 0.026 |
| ≥35 | 109 (9.7%) | 1060 (11.4%) | 154 (13.3%) |
| BMI at delivery (kg/m2) ^a^ | 28.1 ± 3.5 | 27.4 ± 3.4 | 26.3 ± 3.1 | <0.001 |
| <25 | 192 (17.3%) | 2250 (24.5%) | 416 (36.3%) | <0.001 |
| ≥25 | 920 (82.7%) | 6930 (75.5%) | 729 (63.7%) |
| Systolic BP at delivery (mmHg) | 122.0 ± 12.6 | 121.0 ± 12.0 | 119.3 ± 11.2 | <0.001 |
| Diastolic BP at delivery(mmHg) | 75.3 ± 9.0 | 74.5 ± 8.2 | 74.0 ± 7.8 | 0.004 |
| Gravidity |  | | | |
| <3 | 737 (65.7%) | 6614 (71.3%) | 861 (74.5%) | <0.001 |
| ≥3 | 385 (34.3%) | 2660 (28.7%) | 295 (25.5%) |
| Parity |  | | | |
| No child | 563 (50.2%) | 5616 (60.6%) | 756 (65.4%) | <0.001 |
| ≥1 child | 559 (49.8%) | 3658 (39.4%) | 400 (34.6%) |
| Gestational age at delivery (week) | 38.7 ± 1.7 | 38.7 ± 1.7 | 38.6 ± 1.6 | 0.023 |
| Assisted reproduction | 7 (0.6%) | 213 (2.3%) | 44 (3.8%) | <0.001 |
| Pregnancy complication ^b^ |  |  |  |  |
| GDM | 38 (3.4%) | 779 (8.4%) | 148 (12.8%) | <0.001 |
| ICP | 74 (6.6%) | 527 (5.7%) | 110 (9.5%) | <0.001 |
| PE | 62 (5.5%) | 299 (3.2%) | 33 (2.9%) | <0.001 |
| PIH | 28 (2.5%) | 203 (2.2%) | 14 (1.2%) | 0.062 |
| Delivery mode |  | | | |
| Vaginal delivery | 707 (63.0%) | 5319 (57.4%) | 617 (53.4%) | <0.001 |
| Cesarean section | 415 (37.0%) | 3955 (42.6%) | 539 (46.6%) |
| Delivery season |  |  |  |  |
| Spring | 265 (23.6%) | 2043 (22.0%) | 243 (21.0%) | 0.115 |
| Summer | 250 (22.3%) | 2341 (25.2%) | 308 (26.6%) |
| Autumn | 339 (30.2%) | 2555 (27.6%) | 327 (28.3%) |
| Winter | 268 (23.9%) | 2335 (25.2%) | 278 (24.0%) |
| PTB | 88 (7.8%) | 615 (6.6%) | 87 (7.5%) | 0.196 |
| Fetal sex |  |  |  |  |
| Female | 556 (49.6%) | 4374 (47.2%) | 514 (44.5%) | 0.051 |
| Male | 566 (50.4%) | 4900 (52.8%) | 642 (55.5%) |
| Fetal birth length (cm) | 49.8 ± 1.5 | 49.8 ± 1.4 | 49.7 ± 1.5 | 0.004 |
| Fetal birth weight (g) | 3380 (3070–3680) | 3360 (3080–3660) | 3280 (3000–3550) | <0.001 |
| SGA | 96 (8.6%) | 805 (8.7%) | 121 (10.5%) | <0.001 |
| AGA | 835 (74.4%) | 6999 (75.5%) | 905 (78.3%) |
| LGA | 191 (17.0%) | 1470 (15.9%) | 130 (11.2%) |
| **Notes:** Data were presented as median (IQR); mean ±SD and N (%) for continuous variables with normal distribution; continuous variables with skewed distribution; and categorical variables; respectively.  ^a^ 112 cases missing maternal height or weight at delivery.  ^b^ 221 cases had more than one kind of complications.  **Abbreviations:** IQR; interquartile range; SD; standard deviation; P; percentile; BMI; body mass index; BP; blood pressure; GDM, gestational diabetes mellitus; ICP, intrahepatic cholestasis of pregnancy; PE, preeclampsia; PIH, pregnancy-induced hypertension; PTB, pre-term birth; SGA/AGA/LGA, small/appropriate/large for gestational age | | | | |

| **Table S3** Characteristics of study populations according to the categories of maternal serum folate levels during late pregnancy | | | | |
| --- | --- | --- | --- | --- |
|  | Folate levels (μg/L) | | | *P* value |
|  | Low (<P10; N=1155) | Normal (P10-P90; N=9379) | High (>P95; N=1016) |
| Maternal age at delivery (years) | 27.0 ± 4.5 | 28.7 ± 4.4 | 29.7 ± 4.5 | <0.001 |
| <35 | 1080 (93.5%) | 8300 (88.5%) | 847 (83.4%) | <0.001 |
| ≥35 | 75 (6.5%) | 1079 (11.5%) | 169 (16.6%) |
| BMI at delivery (kg/m2) ^a^ | 27.6 ± 3.7 | 27.3 ± 3.3 | 27.0 ± 3.3 | 0.001 |
| <25 | 276 (24.3%) | 2307 (24.8%) | 275 (27.3%) | 0.194 |
| ≥25 | 858 (75.7%) | 6987 (75.2%) | 732 (72.7%) |
| Systolic BP at delivery (mmHg) | 122.4 ± 12.2 | 120.9 ± 12.0 | 120.0 ± 11.8 | <0.001 |
| Diastolic BP at delivery(mmHg) | 75.6 ± 8.4 | 74.4 ± 8.2 | 74.1 ± 8.2 | <0.001 |
| Gravidity |  | | | |
| <3 | 797 (69.0%) | 6647 (70.9%) | 766 (75.4%) | 0.003 |
| ≥3 | 358 (31.0%) | 2732 (29.1%) | 250 (24.6%) |
| Parity |  | | | |
| No child | 643 (55.7%) | 5621 (59.9%) | 669 (65.8%) | <0.001 |
| ≥1 child | 512 (44.3%) | 3758 (40.1%) | 347 (34.2%) |
| Gestational age at delivery (week) | 38.6 ± 1.9 | 38.7 ± 1.6 | 38.5 ± 1.6 | 0.003 |
| Assisted reproduction | 9 (0.8%) | 205 (2.2%) | 50 (4.9%) | <0.001 |
| Pregnancy complication ^b^ |  |  |  |  |
| GDM | 40 (3.5%) | 800 (8.5%) | 125 (12.3%) | <0.001 |
| ICP | 89 (7.7%) | 568 (6.1%) | 54 (5.3%) | 0.045 |
| PE | 58 (5.0%) | 300 (3.2%) | 36 (3.5%) | 0.005 |
| PIH | 30 (2.6%) | 196 (2.1%) | 19 (1.9%) | 0.446 |
| Delivery mode |  | | | |
| Vaginal delivery | 736 (63.7%) | 5422 (57.8%) | 483 (47.5%) | <0.001 |
| Cesarean section | 419 (36.3%) | 3957 (42.2%) | 533 (52.5%) |
| Delivery season |  |  |  |  |
| Spring | 203 (17.6%) | 2189 (23.3%) | 158 (15.6%) | <0.001 |
| Summer | 326 (28.2%) | 2347 (25.0%) | 225 (22.1%) |
| Autumn | 376 (32.6%) | 2491 (26.6%) | 353 (34.7%) |
| Winter | 250 (21.6%) | 2352 (25.1%) | 280 (27.6%) |
| PTB | 117 (10.1%) | 603 (6.4%) | 71 (7.0%) | <0.001 |
| Fetal sex |  |  |  |  |
| Female | 536 (46.4%) | 4401 (46.9%) | 506 (49.8%) | 0.191 |
| Male | 536 (46.4%) | 4401 (46.9%) | 506 (49.8%) |
| Fetal birth length (cm) | 49.7 ± 1.7 | 49.8 ± 1.4 | 49.8 ± 1.5 | 0.037 |
| Fetal birth weight (g) | 3330 (3030–3645) | 3360 (3070–3650) | 3350 (3070–3630) | 0.044 |
| SGA | 120 (10.4%) | 820 (8.7%) | 82 (8.1%) | 0.308 |
| AGA | 867 (75.1%) | 7094 (75.6%) | 776 (76.4%) |
| LGA | 168 (14.5%) | 1465 (15.6%) | 158 (15.6%) |
| **Notes:** Data were presented as median (IQR); mean ±SD and N (%) for continuous variables with normal distribution; continuous variables with skewed distribution; and categorical variables; respectively.  ^a^ 112 cases missing maternal height or weight at delivery.  ^b^ 221 cases had more than one kind of complications  **Abbreviations:** IQR; interquartile range; SD; standard deviation; P; percentile; BMI; body mass index; BP; blood pressure; GDM, gestational diabetes mellitus; ICP, intrahepatic cholestasis of pregnancy; PE, preeclampsia; PIH, pregnancy-induced hypertension; PTB, pre-term birth; SGA/AGA/LGA, small/appropriate/large for gestational age | | | | |

| **Table S4** Adverse pregnancy outcomes | | | | | | |
| --- | --- | --- | --- | --- | --- | --- |
|  | Rate ratio in high SFVB12R group  (95% CI) | Rate ratio in low SFVB12R group  (95% CI) | Rate ratio in  high SF group  (95% CI) | Rate ratio in  low SF group  (95% CI) | Rate ratio in high SVB12 group  (95% CI) | Rate ratio in low SVB12 group  (95% CI) |
| Outcomes |  |  |  |  |  |  |
| GDM | 0.99 (0.69, 1.28) | 0.95 (0.68, 1.24) | 1.50 (1.24, 1.79) | 0.29 (0.15, 0.45) | 1.65 (1.30, 2.00) | 0.38 (0.23, 0.59) |
| ICP | 1.00 (0.67, 1.36) | 1.90 (1.49, 2.37) | 0.90 (0.67, 1.21) | 1.30 (0.95, 1.67) | 1.96 (1.52, 2.50) | 1.23 (0.88, 1.62) |
| PE | 1.23 (0.78, 1.77) | 1.20 (0.72, 1.70) | 1.23 (0.87, 1.69) | 1.77 (1.20, 2.47) | 1.26 (0.75, 1.83) | 1.48 (0.94, 2.04) |
| PIH | 0.74 (0.31, 1.33) | 1.56 (0.91, 2.36) | 1.03 (0.61, 1.55) | 1.06 (0.50, 1.68) | 0.64 (0.20, 1.18) | 1.41 (0.77, 2.11) |
| PTB | 1.17 (0.85, 1.51) | 1.43 (1.09, 1.79) | 1.09 (0.84, 1.35) | 1.52 (1.14, 1.91) | 1.44 (1.09, 1.86) | 1.10 (0.78, 1.44) |
| SGA | 0.78 (0.53, 1.02) | 1.37 (1.08, 1.68) | 0.88 (0.68, 1.09) | 1.28 (1.00, 1.59) | 1.37 (1.06, 1.69) | 1.10 (0.82, 1.44) |
| LGA | 1.35 (1.15, 1.59) | 0.74 (0.56, 0.89) | 0.96 (0.81, 1.12) | 0.93 (0.74, 1.12) | 0.68 (0.50, 0.84) | 1.15 (0.95, 1.35) |
| **Abbreviations:** SFVB12R, serum folate to vitamin B12 ratio; SF, serum folate; SVB12; serum vitamin B12; GDM, gestational diabetes mellitus; ICP, intrahepatic cholestasis of pregnancy; PE, preeclampsia; PIH, pregnancy-induced hypertension; PTB, pre-term birth; SGA/LGA, small/large for gestational age; | | | | | | |

| **Table S5** β (95% CI) for fetal development associated with categories of maternal SF, SVB12 levels and their ratio. | | | | | | |
| --- | --- | --- | --- | --- | --- | --- |
|  | Gestational age (weeks) | | Birth weight (g) | | Birth length (cm) | |
|  | β (95%CI) | *P* value | β (95%CI) | *P* value | β (95%CI) | *P* value |
| Unadjusted |  |  |  |  |  |  |
| SFVB12R |  |  |  |  |  |  |
| P5-P95 | Ref. |  | Ref. |  | Ref. |  |
| > P95 | -0.13 (-0.27, 0.01) | 0.062 | 59.73 (18.38, 101.08) | 0.005 | 0.04 (-0.07, 0.16) | 0.463 |
| < P5 | -0.23 (-0.37, -0.09) | 0.002 | -110.37 (-151.84, -68.90) | <0.001 | -0.13 (-0.25, -0.01) | 0.034 |
| P10-P90 | Ref. |  | Ref. |  | Ref. |  |
| > P90 | -0.01 (-0.11, 0.09) | 0.820 | 54.40 (24.28, 84.51) | <0.001 | 0.10 (0.01, 0.18) | 0.028 |
| < P10 | -0.19 (-0.30, -0.09) | <0.001 | -77.13 (-107.58, -46.67) | <0.001 | -0.09 (-0.18, -0.01) | 0.037 |
| Q1 | Ref. |  | Ref. |  | Ref. |  |
| Q2 | 0.11 (0.02, 0.19) | 0.013 | 35.74 (10.19, 61.28) | 0.006 | 0.02 (-0.05, 0.09) | 0.592 |
| Q3 | 0.14 (0.05, 0.22) | 0.002 | 52.71 (27.16, 78.25) | <0.001 | 0.11 (0.03, 0.18) | 0.004 |
| Q4 | 0.07 (-0.01, 0.16) | 0.104 | 81.05 (55.51, 106.60) | <0.001 | 0.11 (0.03, 0.18) | 0.004 |
| *P* for trend |  | 0.240 |  | <0.001 |  | 0.001 |
| SF |  |  |  |  |  |  |
| P5-P95 | Ref. |  | Ref. |  | Ref. |  |
| > P95 | -0.17 (-0.28, -0.05) | 0.004 | -24.96 (-58.62, 8.71) | 0.146 | -0.02 (-0.12, 0.08) | 0.690 |
| < P5 | -0.13 (-0.27, 0.01) | 0.079 | -60.84 (-102.65, -19.04) | 0.004 | -0.16 (-0.28, -0.04) | 0.010 |
| P10-P90 | Ref. |  | Ref. |  | Ref. |  |
| > P90 | -0.17 (-0.27, -0.06) | 0.002 | -20.11 (-51.94, 11.73) | 0.216 | -0.03 (-0.12, 0.06) | 0.574 |
| < P10 | -0.09 (-0.20, 0.01) | 0.070 | -39.43 (-69.79, -9.07) | 0.011 | -0.14 (-0.22, -0.05) | 0.002 |
| Q1 | Ref. |  | Ref. |  | Ref. |  |
| Q2 | 0.11 (0.02, 0.19) | 0.015 | 33.32 (7.73, 58.90) | 0.011 | 0.11 (0.04, 0.19) | 0.003 |
| Q3 | 0.16 (0.07, 0.24) | <0.001 | 51.73 (26.14, 77.33) | <0.001 | 0.11 (0.04, 0.19) | 0.003 |
| Q4 | -0.01 (-0.10, 0.07) | 0.779 | 34.35 (8.75, 59.95) | 0.009 | 0.10 (0.03, 0.17) | 0.008 |
| *P* for trend |  | 0.231 |  | 0.041 |  | 0.068 |
| SVB12 |  |  |  |  |  |  |
| P5-P95 | Ref. |  | Ref. |  | Ref. |  |
| > P95 | -0.26 (-0.40, -0.12) | <0.001 | -138.61 (-180.25, -96.97) | <0.001 | -0.22 (-0.34, -0.10) | <0.001 |
| < P5 | 0.10 (-0.05, 0.24) | 0.184 | 22.87 (-19.19, 64.92) | 0.287 | 0.03 (-0.09, 0.15) | 0.660 |
| P10-P90 | Ref. |  | Ref. |  | Ref. |  |
| > P90 | -0.14 (-0.24, -0.04) | 0.007 | -86.29 (-116.46, -56.12) | <0.001 | -0.08 (-0.17, 0.00) | 0.064 |
| < P10 | 0.01 (-0.09, 0.12) | 0.810 | 13.84 (-17.04, 44.72) | 0.380 | -0.02 (-0.11, 0.07) | 0.621 |
| Q1 | Ref. |  | Ref. |  | Ref. |  |
| Q2 | 0.06 (-0.02, 0.15) | 0.143 | 2.94 (-22.71, 28.59) | 0.822 | 0.08 (0.00, 0.15) | 0.046 |
| Q3 | 0.01 (-0.07, 0.10) | 0.769 | -16.25 (-41.77, 9.27) | 0.212 | 0.04 (-0.03, 0.12) | 0.231 |
| Q4 | -0.09 (-0.18, -0.01) | 0.034 | -61.59 (-87.11, -36.07) | <0.001 | -0.03 (-0.10, 0.05) | 0.487 |
| *P* for trend |  | 0.006 |  | <0.001 |  | 0.204 |
| Adjusted |  |  |  |  |  |  |
| SFVB12R ^a^ |  |  |  |  |  |  |
| P5-P95 | Ref. |  | Ref. |  | Ref. |  |
| > P95 | -0.14 (-0.27, 0.00) | 0.045 | 60.99 (29.52, 92.45) | <0.001 | 0.07 (-0.02, 0.16) | 0.118 |
| < P5 | -0.18 (-0.32, -0.05) | 0.008 | -43.81 (-75.62, -12.00) | 0.007 | 0.02 (-0.07, 0.12) | 0.594 |
| P10-P90 | Ref. |  | Ref. |  | Ref. |  |
| > P90 | -0.03 (-0.13, 0.06) | 0.507 | 39.45 (16.45, 62.44) | <0.001 | 0.07 (0.00, 0.13) | 0.046 |
| < P10 | -0.14 (-0.24, -0.04) | 0.006 | -25.24 (-48.53, -1.94) | 0.034 | 0.03 (-0.03, 0.10) | 0.317 |
| Q1 | Ref. |  | Ref. |  | Ref. |  |
| Q2 | 0.09 (0.00, 0.17) | 0.039 | 4.28 (-15.21, 23.76) | 0.667 | -0.06 (-0.11, -0.00) | 0.041 |
| Q3 | 0.10 (0.02, 0.19) | 0.014 | 13.21 (-6.35, 32.78) | 0.186 | -0.01 (-0.06, 0.05) | 0.845 |
| Q4 | 0.04 (-0.04, 0.12) | 0.348 | 40.94 (21.27, 60.61) | <0.001 | 0.01 (-0.05, 0.06) | 0.774 |
| *P* for trend |  | 0.614 |  | <0.001 |  | 0.277 |
| SF ^b^ |  |  |  |  |  |  |
| P5-P95 | Ref. |  | Ref. |  | Ref. |  |
| > P95 | -0.12 (-0.23, -0.01) | 0.037 | 19.08 (-7.03, 45.18) | 0.152 | 0.06 (-0.01, 0.14) | 0.090 |
| < P5 | -0.07 (-0.21, 0.07) | 0.301 | -29.25 (-61.74, 3.25) | 0.078 | -0.03 (-0.12, 0.06) | 0.508 |
| P10-P90 | Ref. |  | Ref. |  | Ref. |  |
| > P90 | -0.12 (-0.23, -0.02) | 0.022 | 23.49 (-1.35, 48.34) | 0.064 | 0.06 (-0.01, 0.13) | 0.096 |
| < P10 | -0.05 (-0.15, 0.05) | 0.334 | -20.63 (-44.48, 3.23) | 0.090 | -0.04 (-0.11, 0.03) | 0.266 |
| Q1 | Ref. |  | Ref. |  | Ref. |  |
| Q2 | 0.10 (0.02, 0.19) | 0.015 | 14.30 (-5.34, 33.94) | 0.154 | 0.05 (-0.01, 0.10) | 0.114 |
| Q3 | 0.16 (0.07, 0.24) | <0.001 | 26.23 (6.24, 46.22) | 0.010 | 0.00 (-0.06, 0.05) | 0.934 |
| Q4 | 0.02 (-0.07, 0.10) | 0.719 | 46.79 (25.88, 67.69) | <0.001 | 0.07 (0.01, 0.13) | 0.029 |
| *P* for trend |  | 0.581 |  | <0.001 |  | 0.074 |
| SVB12 ^c^ |  |  |  |  |  |  |
| P5-P95 | Ref. |  | Ref. |  | Ref. |  |
| > P95 | -0.20 (-0.34, -0.06) | 0.004 | -61.87 (-94.26, -29.47) | <0.001 | -0.08 (-0.17, 0.01) | 0.093 |
| < P5 | 0.12 (-0.01, 0.26) | 0.077 | 6.33 (-26.18, 38.84) | 0.703 | 0.00 (-0.09, 0.09) | 0.983 |
| P10-P90 | Ref. |  | Ref. |  | Ref. |  |
| > P90 | -0.10 (-0.20, -0.00) | 0.041 | -42.99 (-66.70, -19.29) | <0.001 | -0.02 (-0.09, 0.05) | 0.607 |
| < P10 | 0.03 (-0.07, 0.14) | 0.511 | 8.89 (-15.15, 32.93) | 0.469 | 0.00 (-0.07, 0.06) | 0.915 |
| Q1 | Ref. |  | Ref. |  | Ref. |  |
| Q2 | 0.06 (-0.02, 0.14) | 0.169 | 0.93 (-18.82, 20.67) | 0.927 | 0.04 (-0.02, 0.09) | 0.205 |
| Q3 | 0.02 (-0.06, 0.11) | 0.597 | -4.40 (-24.47, 15.67) | 0.667 | 0.05 (-0.01, 0.10) | 0.117 |
| Q4 | -0.07 (-0.16, 0.02) | 0.123 | -27.18 (-48.16, -6.19) | 0.011 | 0.02 (-0.04, 0.08) | 0.578 |
| *P* for trend |  | 0.039 |  | 0.005 |  | 0.730 |
| **Notes:** ^a^ β values for gestational age were adjusted for maternal age, BMI, gravidity, parity, blood pressure, GDM, ICP, PE, PIH, and fetal sex; β values for birthweight and birth length were additionally adjusted for gestational age. ^b^ additionally adjusted for SVB12 levels. ^c^ additionally adjusted for SF levels.  **Abbreviations:** OR, odds ratio; CI, confidence interval; SFVB12R, serum folate to vitamin B12 ratio; P; percentile; Q, quartile; SF, serum folate, SVB12, serum vitamin B12; GDM, gestational diabetes mellitus; ICP, intrahepatic cholestasis of pregnancy; PE, pre-eclampsia; PIH, pregnancy induced hypertension; BMI, body mass index. | | | | | | |

| **Table S6** ORs and 95% CIs for different pregnancy complications with categories of maternal SF, SVB12 levels and their ratio. | | | | | | | | |
| --- | --- | --- | --- | --- | --- | --- | --- | --- |
|  |  |  |  |  |  |  |  |  |
|  | GDM | | ICP | | PE | | PIH | |
|  | OR (95%CI) | *P* value | OR (95%CI) | *P* value | OR (95%CI) | *P* value | OR (95%CI) | *P* value |
| Unadjusted |  | | | | | | | |
| SFVB12R |  |  |  |  |  |  |  |  |
| P5-P95 | Ref. |  | Ref. |  | Ref. |  | Ref. |  |
| > P95 | 0.98 (0.73, 1.34) | 0.920 | 1.00 (0.70, 1.42) | 0.982 | 1.24 (0.81, 1.89) | 0.327 | 0.74 (0.38, 1.45) | 0.381 |
| < P5 | 0.94 (0.68, 1.30) | 0.712 | 2.03 (1.55, 2.67) | <0.001 | 1.21 (0.78, 1.88) | 0.398 | 1.58 (0.97, 2.58) | 0.067 |
| P10-P90 | Ref. |  | Ref. |  | Ref. |  | Ref. |  |
| > P90 | 1.08 (0.87, 1.34) | 0.500 | 0.90 (0.68, 1.19) | 0.453 | 0.98 (0.70, 1.39) | 0.926 | 0.92 (0.59, 1.44) | 0.730 |
| < P10 | 0.93 (0.74, 1.17) | 0.535 | 1.81 (1.47, 2.24) | <0.001 | 1.24 (0.90, 1.71) |  | 1.40 (0.96, 2.05) | 0.082 |
| Q1 | Ref. |  | Ref. |  | Ref. |  | Ref. |  |
| Q2 | 1.03 (0.84, 1.25) | 0.791 | 0.71 (0.58, 0.87) | <0.001 | 0.91 (0.69, 1.19) | 0.485 | 0.87 (0.61, 1.25) | 0.449 |
| Q3 | 1.15 (0.95, 1.40) | 0.143 | 0.63 (0.51, 0.78) | <0.001 | 0.70 (0.52, 0.93) | 0.015 | 1.01 (0.71, 1.43) | 0.957 |
| Q4 | 1.28 (1.06, 1.54) | 0.011 | 0.57 (0.46, 0.70) | <0.001 | 0.77 (0.58, 1.02) | 0.066 | 0.89 (0.62, 1.27) | 0.522 |
| *P* for trend |  | 0.004 |  | <0.001 |  | 0.040 |  | 0.698 |
| SF |  |  |  |  |  |  |  |  |
| P5-P95 | Ref. |  | Ref. |  | Ref. |  | Ref. |  |
| > P95 | 1.58 (1.28, 1.95) | <0.001 | 0.90 (0.66, 1.22) | 0.488 | 1.24 (0.87, 1.77) | 0.235 | 1.03 (0.64, 1.65) | 0.909 |
| < P5 | 0.27 (0.16, 0.47) | <0.001 | 1.33 (0.97, 1.81) | 0.074 | 1.82 (1.26, 2.62) | 0.001 | 1.06 (0.60, 1.87) | 0.836 |
| P10-P90 | Ref. |  | Ref. |  | Ref. |  | Ref. |  |
| > P90 | 1.48 (1.21, 1.81) | <0.001 | 0.90 (0.67, 1.20) | 0.465 | 1.13 (0.80, 1.61) | 0.487 | 0.92 (0.57, 1.47) | 0.717 |
| < P10 | 0.40 (0.29, 0.56) | <0.001 | 1.26 (1.00, 1.60) | 0.049 | 1.56 (1.17, 2.08) | 0.003 | 1.23 (0.84, 1.82) | 0.289 |
| Q1 | Ref. |  | Ref. |  | Ref. |  | Ref. |  |
| Q2 | 1.33 (1.08, 1.65) | 0.009 | 0.92 (0.75, 1.13) | 0.439 | 0.87 (0.67, 1.13) | 0.311 | 0.94 (0.67, 1.33) | 0.735 |
| Q3 | 1.60 (1.30, 1.97) | <0.001 | 0.80 (0.65, 0.99) | 0.040 | 0.55 (0.40, 0.74) | <0.001 | 0.84 (0.59, 1.19) | 0.324 |
| Q4 | 2.31 (1.89, 2.81) | <0.001 | 0.80 (0.64, 0.99) | 0.043 | 0.73 (0.55, 0.96) | 0.024 | 0.79 (0.55, 1.13) | 0.202 |
| *P* for trend |  |  |  | 0.039 |  |  |  | 0.181 |
| SVB12 |  |  |  |  |  |  |  |  |
| P5-P95 | Ref. |  | Ref. |  | Ref. |  | Ref. |  |
| > P95 | 1.77 (1.37, 2.27) | <0.001 | 2.11 (1.60, 2.78) | <0.001 | 1.28 (0.82, 1.99) | 0.278 | 0.63 (0.30, 1.35) | 0.236 |
| < P5 | 0.36 (0.22, 0.59) | <0.001 | 1.25 (0.90, 1.74) | 0.182 | 1.51 (1.01, 2.24) | 0.043 | 1.42 (0.86, 2.35) | 0.168 |
| P10-P90 | Ref. |  | Ref. |  | Ref. |  | Ref. |  |
| > P90 | 1.62 (1.34, 1.96) | <0.001 | 1.78 (1.43, 2.21) | <0.001 | 0.94 (0.65, 1.36) | 0.745 | 0.59 (0.34, 1.02) | 0.057 |
| < P10 | 0.40 (0.29, 0.56) | <0.001 | 1.15 (0.89, 1.48) | 0.275 | 1.70 (1.28, 2.25) | <0.001 | 1.13 (0.76, 1.69) | 0.548 |
| Q1 | Ref. |  | Ref. |  | Ref. |  | Ref. |  |
| Q2 | 1.60 (1.28, 1.99) | <0.001 | 1.05 (0.84, 1.31) | 0.682 | 0.77 (0.58, 1.01) | 0.058 | 1.11 (0.78, 1.59) | 0.550 |
| Q3 | 1.94 (1.56, 2.39) | <0.001 | 1.09 (0.87, 1.36) | 0.444 | 0.75 (0.57, 0.99) | 0.039 | 0.98 (0.68, 1.41) | 0.919 |
| Q4 | 2.62 (2.13, 3.21) | <0.001 | 1.37 (1.11, 1.69) | 0.004 | 0.68 (0.51, 0.90) | 0.008 | 1.09 (0.76, 1.56) | 0.638 |
| *P* for trend |  | <0.001 |  | 0.002 |  | 0.011 |  | 0.782 |
| Adjusted |  |  |  |  |  |  |  |  |
| SFVB12R ^a^ |  |  |  |  |  |  |  |  |
| P5-P95 | Ref. |  | Ref. |  | Ref. |  | Ref. |  |
| > P95 | 0.82 (0.60, 1.13) | 0.230 | 0.99 (0.69, 1.42) | 0.962 | 0.99 (0.63, 1.54) | 0.948 | 0.68 (0.34, 1.34) | 0.261 |
| < P5 | 1.05 (0.75, 1.47) | 0.768 | 2.03 (1.54, 2.67) | <0.001 | 1.29 (0.80, 2.07) | 0.303 | 1.81 (1.09, 3.00) | 0.021 |
| P10-P90 | Ref. |  | Ref. |  | Ref. |  | Ref. |  |
| > P90 | 0.92 (0.74, 1.15) | 0.485 | 0.87 (0.66, 1.15) | 0.330 | 0.82 (0.57, 1.17) | 0.267 | 0.84 (0.53, 1.32) | 0.441 |
| < P10 | 1.05 (0.83, 1.34) | 0.679 | 1.83 (1.47, 2.26) | <0.001 | 1.35 (0.97, 1.89) | 0.075 | 1.59 (1.07, 2.35) | 0.020 |
| Q1 | Ref. |  | Ref. |  | Ref. |  | Ref. |  |
| Q2 | 0.96 (0.78, 1.17) | 0.664 | 0.70 (0.57, 0.86) | <0.001 | 0.84 (0.64, 1.11) | 0.230 | 0.80 (0.55, 1.15) | 0.230 |
| Q3 | 1.02 (0.84, 1.24) | 0.849 | 0.62 (0.50, 0.77) | <0.001 | 0.63 (0.47, 0.85) | 0.003 | 0.92 (0.64, 1.31) | 0.641 |
| Q4 | 1.01 (0.83, 1.23) | 0.922 | 0.54 (0.44, 0.68) | <0.001 | 0.61 (0.45, 0.81) | <0.001 | 0.72 (0.50, 1.04) | 0.083 |
| *P* for trend |  | 0.752 |  | <0.001 |  | <0.001 |  | 0.147 |
| SF ^b^ |  | | | | | | | |
| P5-P95 | Ref. |  | Ref. |  | Ref. |  | Ref. |  |
| > P95 | 1.23 (0.99, 1.53) | 0.065 | 0.76 (0.55, 1.03) | 0.077 | 1.14 (0.79, 1.67) | 0.482 | 1.05 (0.65, 1.72) | 0.835 |
| < P5 | 0.40 (0.23, 0.70) | 0.001 | 1.58 (1.15, 2.17) | 0.005 | 1.89 (1.28, 2.81) | 0.002 | 1.11 (0.62, 2.00) | 0.718 |
| P10-P90 | Ref. |  | Ref. |  | Ref. |  | Ref. |  |
| > P90 | 1.17 (0.94, 1.44) |  | 0.75 (0.55, 1.00) | 0.053 | 1.03 (0.71, 1.50) | 0.874 | 0.93 (0.57, 1.52) | 0.775 |
| < P10 | 0.55 (0.39, 0.77) | <0.001 | 1.44 (1.13, 1.84) | 0.003 | 1.57 (1.15, 2.14) | 0.005 | 1.21 (0.80, 1.83) | 0.366 |
| Q1 | Ref. |  | Ref. |  | Ref. |  | Ref. |  |
| Q2 | 1.16 (0.93, 1.45) | 0.183 | 0.83 (0.68, 1.03) | 0.092 | 0.83 (0.63, 1.09) | 0.182 | 0.93 (0.65, 1.32) | 0.692 |
| Q3 | 1.27 (1.03, 1.58) | 0.028 | 0.68 (0.55, 0.85) | <0.001 | 0.52 (0.38, 0.71) | <0.001 | 0.82 (0.57, 1.18) | 0.283 |
| Q4 | 1.53 (1.24, 1.90) | <0.001 | 0.60 (0.48, 0.76) | <0.001 | 0.63 (0.46, 0.86) | 0.003 | 0.68 (0.46, 1.01) | 0.059 |
| *P* for trend |  | <0.001 |  | <0.001 |  | 0.003 |  | 0.047 |
| SVB12 ^c^ |  | | | | | | | |
| P5-P95 | Ref. |  | Ref. |  | Ref. |  | Ref. |  |
| > P95 | 1.62 (1.24, 2.11) | <0.001 | 2.22 (1.67, 2.96) | <0.001 | 1.69 (1.04, 2.74) | 0.033 | 0.88 (0.40, 1.90) | 0.740 |
| < P5 | 0.41 (0.25, 0.69) | 0.001 | 1.23 (0.88, 1.72) | 0.227 | 1.22 (0.80, 1.87) | 0.351 | 1.32 (0.79, 2.21) | 0.296 |
| P10-P90 | Ref. |  | Ref. |  | Ref. |  | Ref. |  |
| > P90 | 1.49 (1.22, 1.82) | <0.001 | 1.88 (1.49, 2.36) | <0.001 | 1.18 (0.79, 1.74) | 0.420 | 0.78 (0.44, 1.36) | 0.375 |
| < P10 | 0.45 (0.32, 0.63) | <0.001 | 1.11 (0.86, 1.44) | 0.411 | 1.35 (1.00, 1.84) | 0.051 | 1.01 (0.66, 1.53) | 0.979 |
| Q1 | Ref. |  | Ref. |  | Ref. |  | Ref. |  |
| Q2 | 1.53 (1.22, 1.92) | <0.001 | 1.05 (0.83, 1.31) | 0.690 | 0.87 (0.66, 1.16) | 0.342 | 1.33 (0.92, 1.91) | 0.132 |
| Q3 | 1.78 (1.43, 2.23) | <0.001 | 1.12 (0.89, 1.40) | 0.341 | 0.92 (0.69, 1.23) | 0.558 | 1.19 (0.81, 1.74) | 0.386 |
| Q4 | 2.33 (1.86, 2.91) | <0.001 | 1.46 (1.16, 1.84) | 0.001 | 0.96 (0.70, 1.31) | 0.777 | 1.59 (1.07, 2.36) | 0.021 |
| *P* for trend |  | <0.001 |  | <0.001 |  | 0.871 |  | 0.038 |
| **Notes:** ^a^ OR values were adjusted for maternal age, BMI, gravidity, parity. ^b^ additionally adjusted for SVB12 levels. ^c^ additionally adjusted for SF levels.  **Abbreviations:** OR, odds ratio; CI, confidence interval; SFVB12R, serum folate to vitamin B12 ratio; P; percentile; Q, quartile; SF, serum folate, SVB12, serum vitamin B12; GDM, gestational diabetes mellitus; ICP, intrahepatic cholestasis of pregnancy; PE, pre-eclampsia; PIH, pregnancy induced hypertension; BMI, body mass index. | | | | | | | | |
|  |  |  |  |  |  |  |  |  |

| **Table S7** ORs and 95% CIs for adverse birth outcomes with maternal SF, SVB12 levels and their ratio | | | | | | | |
| --- | --- | --- | --- | --- | --- | --- | --- |
|  | | PTB | | SGA | | LGA | |
|  | | OR (95%CI) | *P* value | OR (95%CI) | *P* value | OR (95%CI) | *P* value |
| Unadjusted | |  |  |  |  |  |  |
| SFVB12R | |  |  |  |  |  |  |
| P5-P95 | | Ref. |  | Ref. |  | Ref. |  |
| > P95 | | 1.18 (0.86, 1.62) | 0.301 | 0.76 (0.54, 1.07) | 0.118 | 1.45 (1.18, 1.78) | <0.001 |
| < P5 | | 1.48 (1.11, 1.97) | 0.008 | 1.43 (1.10, 1.84) | 0.007 | 0.70 (0.53, 0.92) | 0.009 |
| P10-P90 | | Ref. |  | Ref. |  | Ref. |  |
| > P90 | | 0.93 (0.72, 1.20) | 0.565 | 0.71 (0.55, 0.91) | 0.008 | 1.20 (1.02, 1.41) | 0.026 |
| < P10 | | 1.49 (1.21, 1.85) | <0.001 | 1.35 (1.11, 1.64) | 0.003 | 0.90 (0.76, 1.08) | 0.265 |
| Q1 | | Ref. |  | Ref. |  | Ref. |  |
| Q2 | | 0.87 (0.72, 1.06) | 0.164 | 0.91 (0.77, 1.09) | 0.299 | 1.03 (0.89, 1.19) | 0.710 |
| Q3 | | 0.74 (0.61, 0.91) | 0.004 | 0.81 (0.68, 0.97) | 0.021 | 1.03 (0.89, 1.19) | 0.699 |
| Q4 | | 0.73 (0.60, 0.90) | 0.003 | 0.68 (0.56, 0.82) | <0.001 | 1.20 (1.04, 1.38) | 0.012 |
| *P* for trend | |  | 0.002 |  | <0.001 |  | 0.008 |
| SF | |  |  |  |  |  |  |
| P5-P95 | | Ref. |  | Ref. |  | Ref. |  |
| > P95 | | 1.09 (0.84, 1.42) | 0.514 | 0.86 (0.67, 1.12) | 0.263 | 0.95 (0.79, 1.15) | 0.62 |
| < P5 | | 1.58 (1.19, 2.10) | 0.001 | 1.33 (1.02, 1.74) | 0.038 | 0.92 (0.72, 1.17) | 0.493 |
| P10-P90 | | Ref. |  | Ref. |  | Ref. |  |
| > P90 | | 1.09 (0.85, 1.41) | 0.492 | 0.91 (0.72, 1.16) | 0.461 | 0.99 (0.82, 1.18) | 0.878 |
| < P10 | | 1.64 (1.33, 2.02) | <0.001 | 1.20 (0.98, 1.47) | 0.084 | 0.94 (0.79, 1.12) | 0.475 |
| Q1 | | Ref. |  | Ref. |  | Ref. |  |
| Q2 | | 0.74 (0.61, 0.90) | 0.003 | 0.92 (0.77, 1.10) | 0.378 | 1.15 (0.99, 1.32) | 0.066 |
| Q3 | | 0.67 (0.55, 0.82) | <0.001 | 0.79 (0.66, 0.95) | 0.013 | 1.04 (0.90, 1.21) | 0.597 |
| Q4 | | 0.75 (0.62, 0.92) | 0.005 | 0.79 (0.66, 0.95) | 0.012 | 1.13 (0.98, 1.31) | 0.097 |
| *P* for trend | |  | 0.029 |  | 0.010 |  | 0.283 |
| SVB12 | |  |  |  |  |  |  |
| P5-P95 | | Ref. |  | Ref. |  | Ref. |  |
| > P95 | | 1.49 (1.12, 1.99) | 0.007 | 1.43 (1.10, 1.85) | 0.007 | 0.63 (0.48, 0.84) | 0.001 |
| < P5 | | 1.10 (0.80, 1.53) | 0.55 | 1.12 (0.83, 1.50) | 0.466 | 1.18 (0.94, 1.48) | 0.147 |
| P10-P90 | | Ref. |  | Ref. |  | Ref. |  |
| > P90 | | 1.15 (0.91, 1.45) | 0.253 | 1.16 (0.95, 1.42) | 0.147 | 0.68 (0.56, 0.83) | <0.001 |
| < P10 | | 1.20 (0.95, 1.51) | 0.127 | 1.00 (0.80, 1.25) | 0.997 | 1.09 (0.92, 1.29) | 0.316 |
| Q1 | | Ref. |  | Ref. |  | Ref. |  |
| Q2 | | 0.82 (0.67, 1.01) | 0.063 | 0.96 (0.79, 1.16) | 0.680 | 1.05 (0.91, 1.21) | 0.475 |
| Q3 | | 0.79 (0.65, 0.97) | 0.026 | 1.11 (0.92, 1.33) | 0.280 | 0.96 (0.83, 1.10) | 0.547 |
| Q4 | | 0.94 (0.77, 1.14) | 0.507 | 1.16 (0.96, 1.39) | 0.116 | 0.87 (0.75, 1.00) | 0.054 |
| *P* for trend | |  | 0.701 |  | 0.049 |  | 0.019 |
| Adjusted | |  |  |  |  |  |  |
| SFVB12R ^a^ | |  |  |  |  |  |  |
| P5-P95 | | Ref. |  | Ref. |  | Ref. |  |
| > P95 | | 1.28 (0.92, 1.77) | 0.138 | 0.82 (0.58, 1.16) | 0.26 | 1.32 (1.06, 1.65) | 0.013 |
| < P5 | | 1.33 (0.98, 1.80) | 0.069 | 1.24 (0.95, 1.63) | 0.112 | 0.75 (0.56, 1.00) | 0.049 |
| P10-P90 | | Ref. |  | Ref. |  | Ref. |  |
| > P90 | | 1.02 (0.79, 1.33) | 0.854 | 0.77 (0.59, 0.99) | 0.044 | 1.13 (0.95, 1.33) | 0.173 |
| < P10 | | 1.34 (1.06, 1.68) | 0.013 | 1.22 (0.99, 1.50) | 0.059 | 0.94 (0.77, 1.13) | 0.496 |
| Q1 | | Ref. |  | Ref. |  | Ref. |  |
| Q2 | | 0.93 (0.76, 1.14) | 0.492 | 0.96 (0.80, 1.16) | 0.687 | 0.99 (0.85, 1.15) | 0.880 |
| Q3 | | 0.82 (0.66, 1.02) | 0.071 | 0.89 (0.73, 1.07) | 0.199 | 0.98 (0.84, 1.14) | 0.777 |
| Q4 | | 0.86 (0.69, 1.06) | 0.154 | 0.76 (0.63, 0.93) | 0.007 | 1.09 (0.93, 1.26) | 0.287 |
| *P* for trend | |  | 0.127 |  | 0.004 |  | 0.220 |
| SF ^b^ | |  |  |  |  |  |  |
| P5-P95 | | Ref. |  | Ref. |  | Ref. |  |
| > P95 | | 1.07 (0.81, 1.42) | 0.616 | 0.81 (0.62, 1.06) | 0.118 | 0.99 (0.81, 1.22) | 0.953 |
| < P5 | | 1.27 (0.92, 1.74) | 0.141 | 1.20 (0.90, 1.61) | 0.211 | 0.91 (0.69, 1.18) | 0.458 |
| P10-P90 | | Ref. |  | Ref. |  | Ref. |  |
| > P90 | | 1.06 (0.81, 1.39) | 0.662 | 0.85 (0.66, 1.10) | 0.218 | 1.03 (0.85, 1.25) | 0.791 |
| < P10 | | 1.39 (1.11, 1.76) | 0.005 | 1.12 (0.90, 1.40) | 0.298 | 0.91 (0.75, 1.10) | 0.323 |
| Q1 | | Ref. |  | Ref. |  | Ref. |  |
| Q2 | | 0.78 (0.64, 0.97) | 0.022 | 0.95 (0.79, 1.15) | 0.606 | 1.18 (1.01, 1.38) | 0.032 |
| Q3 | | 0.72 (0.58, 0.90) | 0.003 | 0.82 (0.68, 1.00) | 0.047 | 1.07 (0.91, 1.26) | 0.396 |
| Q4 | | 0.80 (0.64, 1.00) | 0.053 | 0.77 (0.62, 0.94) | 0.010 | 1.20 (1.02, 1.41) | 0.029 |
| *P* for trend | |  | 0.175 |  | 0.006 |  | 0.121 |
| SVB12 ^c^ | |  |  |  |  |  |  |
| P5-P95 | | Ref. |  | Ref. |  | Ref. |  |
| > P95 | | 1.46 (1.07, 1.99) | 0.016 | 1.31 (1.00, 1.73) | 0.053 | 0.70 (0.52, 0.94) | 0.017 |
| < P5 | | 0.97 (0.69, 1.38) | 0.877 | 1.10 (0.80, 1.50) | 0.553 | 1.19 (0.93, 1.51) | 0.159 |
| P10-P90 | | Ref. |  | Ref. |  | Ref. |  |
| > P90 | | 1.16 (0.90, 1.49) | 0.242 | 1.13 (0.91, 1.41) | 0.275 | 0.72 (0.59, 0.89) | 0.002 |
| < P10 | | 1.08 (0.84, 1.39) | 0.546 | 0.95 (0.75, 1.20) | 0.662 | 1.06 (0.88, 1.26) | 0.560 |
| Q1 | | Ref. |  | Ref. |  | Ref. |  |
| Q2 | | 0.85 (0.69, 1.06) | 0.151 | 0.95 (0.78, 1.16) | 0.641 | 1.13 (0.97, 1.31) | 0.121 |
| Q3 | | 0.81 (0.65, 1.01) | 0.061 | 1.10 (0.91, 1.35) | 0.321 | 1.04 (0.89, 1.22) | 0.595 |
| Q4 | | 0.99 (0.80, 1.24) | 0.957 | 1.15 (0.94, 1.41) | 0.181 | 0.96 (0.82, 1.14) | 0.666 |
| *P* for trend | |  | 0.845 |  | 0.086 |  | 0.374 |
|  | **Notes:** ^a^ OR values for gestational age were adjusted for maternal age, BMI, gravidity, parity, blood pressure, GDM, ICP, PE, PIH, and fetal sex; OR values for SGA and LGA were additionally adjusted for gestational age. ^b^ additionally adjusted for SVB12 levels. ^c^ additionally adjusted for SF levels.  **Abbreviations:** OR, odds ratio; CI, confidence interval; PTB, pre-term birth; SGA/LGA, small/large for gestational age; SFVB12R, serum folate to vitamin B12 ratio; P; percentile; Q, quartile; SF, serum folate, SVB12, serum vitamin B12; GDM, gestational diabetes mellitus; ICP, intrahepatic cholestasis of pregnancy; PE, pre-eclampsia; PIH, pregnancy induced hypertension; BMI, body mass index. | | | | | | |
